# Non-coding ribonucleic acid-mediated *CAMSAP1* upregulation leads to poor prognosis with suppressed immune infiltration in liver hepatocellular carcinoma

**DOI:** 10.3389/fgene.2022.916847

**Published:** 2022-09-21

**Authors:** Wenwen Wang, Jingjing Zhang, Yuqing Wang, Yasi Xu, Shirong Zhang

**Affiliations:** Translational Medicine Research Center, Key Laboratory of Clinical Cancer Pharmacology and Toxicology Research of Zhejiang Province, Affiliated Hangzhou First People’s Hospital, Zhejiang University School of Medicine, Cancer Center, Zhejiang University, Hangzhou, China

**Keywords:** CAMSAP1, immune infiltration, hepatocellular carcinoma, non-coding RNA, prognosis

## Abstract

Liver hepatocellular carcinoma (LIHC) is well-known for its unfavorable prognosis due to the lack of reliable diagnostic and prognostic biomarkers. Calmodulin-regulated spectrin-associated protein 1 (*CAMSAP1*) is a non-centrosomal microtubule minus-end binding protein that regulates microtubule dynamics. This study aims to investigate the specific role and mechanisms of *CAMSAP1* in LIHC. We performed systematical analyses of *CAMSAP1* and demonstrated that differential expression of *CAMSAP1* is associated with genetic alteration and DNA methylation, and serves as a potential diagnostic and prognostic biomarker in some cancers, especially LIHC. Further evidence suggested that *CAMSAP1* overexpression leads to adverse clinical outcomes in advanced LIHC. Moreover, the AC145207.5/LINC01748-miR-101–3p axis is specifically responsible for *CAMSAP1* overexpression in LIHC. In addition to the previously reported functions in the cell cycle and regulation of actin cytoskeleton, *CAMSAP1*-related genes are enriched in cancer- and immune-associated pathways. As expected, *CAMSAP1*-associated LIHC is infiltrated in the suppressed immune microenvironment. Specifically, except for immune cell infiltration, it is significantly positively correlated with immune checkpoint genes, especially *CD274* (PD-L1), and cancer-associated fibroblasts. Prediction of immune checkpoint blockade therapy suggests that these patients may benefit from therapy. Our study is the first to demonstrate that besides genetic alteration and DNA methylation, AC145207.5/LINC01748-miR-101-3p-mediated *CAMSAP1* upregulation in advanced LIHC leads to poor prognosis with suppressed immune infiltration, representing a potential diagnostic and prognostic biomarker as well as a promising immunotherapy target for LIHC.

## Introduction

Liver hepatocellular carcinoma (LIHC) is the sixth most commonly diagnosed cancer and the third major cause of cancer-related death worldwide, according to the Global *Cancer* Statistics report 2020 (Sung et al., 2021). Over 910,000 patients develop LIHC every year, and approximately 830,000 patients die from it. The common risk factors for LIHC include chronic viral infection (Pawlotsky, 2004; Trepo et al., 2014), non-alcoholic fatty liver disease (Hindson, 2021), excessive alcohol consumption (Gao and Bataller, 2011), obesity (Lee et al., 2021), aflatoxin exposure (Kimanya et al., 2021), hemochromatosis (Olynyk and Ramm, 2021), primary cholangitis (Lundberg Bave et al., 2021), and immune system diseases (Marcucci and Rumio, 2021). Despite the availability of multiple diagnostic biomarkers for LIHC, their reliability remains questionable, thereby contributing to poor prognosis (Ahn et al., 2021). Therefore, there is an urgent need to identify new biomarkers associated with tumor stage and prognosis to assist the early diagnosis, treatment, and patient management in LIHC.

The microtubule (MT) skeleton is a critical molecular target for tumor therapy (Jordan and Wilson, 2004; Jackson et al., 2007), and MT-associated proteins play essential roles in cell proliferation, polarization, and migration by regulating MT assembly and dynamics (Goodson and Jonasson, 2018). In recent years, as non-centrosomal MT minus binding proteins, members of the Calmodulin-regulated spectrin-associated protein (*CAMSAP*) family, including *CAMSAP1*, *CAMSAP2,* and *CAMSAP3*, have attracted increasing attention for their role in cancers. *CAMSAP1* mutation is a potential prognostic biomarker for small cell lung cancer, indicating platinum sensitivity in lung cancer patients (Yi et al., 2021). Loss of *CAMSAP3* induces senescence in lung cancer cells by regulating the activity of extracellular signal-regulated kinase (Wattanathamsan et al., 2021). *CAMSAP2* expression is upregulated in LIHC and leads to tumor metastasis and poor prognosis (Li D. et al., 2020). Interestingly, different from the entire MT minus binding of CAMSAP2 and CAMSAP3, CAMSAP1 specifically binds to the minus ends of the free or growing MT, protects the minus ends from depolymerization but does not affect the growth rate of the minus, thus accurately regulating MT assembly and dynamics (Baines et al., 2009; Bai et al., 2011; Hendershott and Vale, 2014; Jiang et al., 2014; Richardson et al., 2014; Jiang et al., 2018; Atherton et al., 2019; Chen et al., 2020). CAMSAP1 interacts with Spectrin to regulate neurite outgrowth by regulating the number of MTs (King et al., 2014). *CAMSAP1*-deficient neurons develop multiple axon phenotypes *in vitro*, while the multipolar-bipolar transition and radial migration are blocked *in vivo* (Zhou et al., 2020). In addition, phosphorylation of CAMSAP1 by polarity regulating kinase MARK2 prevents its binding to MTs, altering MT minus-end protection and resulting in asymmetric distribution of MTs (Liu et al., 2020). Moreover, *CAMSAP1* is downregulated by miR-126 upregulation in primary human osteoblasts co-cultured with human umbilical vein endothelial cells, thus promoting the differentiation of 2 cell types (Strassburg et al., 2017); Meanwhile, In laryngeal squamous cell carcinoma, *CAMSAP1* and protein expression are also negatively regulated by miR-126, and loss of miR-126 induces MT formation and aggregation, thus promoting tumor metastasis (Sun et al., 2014). However, the expression, diagnosis, survival, and correlation analyses of *CAMSAP1* with tumor immune infiltration in LIHC have not been comprehensively evaluated.

In this study, we conducted comprehensive pan-cancer analyses of *CAMSAP1* and demonstrated that *CAMSAP1* expression is associated with genetic alteration and DNA methylation. Additionally, AC145207.5/LINC01748-miR-101–3p constitutes an upstream non-coding RNA (ncRNA) axis that regulates *CAMSAP1* expression in LIHC. Overexpressed *CAMSAP1* leads to poor prognosis in advanced LIHC. Finally, the relationship between *CAMSAP1* expression and immune infiltration was investigated. Collectively, we suggest that the ncRNA-mediated *CAMSAP1* overexpression is associated with unfavorable LIHC prognosis with suppressed immune infiltration and immune checkpoint blockade (ICB) treatment may be effective for these patients.

## Materials and methods

### Data acquisition and processing

Transcriptome data including mRNA, microRNA (miRNA), long non-coding RNA (lncRNA), and clinical information were downloaded from The *Cancer* Genome Atlas (TCGA, https://portal.gdc.cancer.gov/repository) and UCSC Xena (https://xenabrowser.net) databases (Liu et al., 2018). Among them, TCGA database contains mRNA and lncRNA sequencing data in level 3 HTSeq-FPKM format, and miRNA sequencing data in level 3 BCGSC format, all of them need to be converted into TPM and RPM format, respectively. UCSC Xena database contains both TCGA and Genotype-Tissue Expression (GTEx) data, which is processed by the Toil process into TPM format (Vivian et al., 2017). GSE45267 dataset comprising 39 normal and 48 LIHC samples were downloaded from the Gene Expression Omnibus (GEO, https://www.ncbi.nlm.nih.gov/geo/) database, and converted into TPM format (Wang et al., 2013). All the TPM, RPM, and clinical data were used for the following analyses, including expression, correlation, diagnoses, and survival analyses. R software (Version 3.6.3) and ggplot2 package (Version 3.3.3) were used for statistical analyses and visualization, respectively.

### Differential expression analysis

Differential expression analysis of *CAMSAP1* was performed using TCGA and/or GTEx databases (Frost et al., 2020; Izzi et al., 2020), and Oncomine data containing 424 sets of analyses were used for further verification (threshold: *p* < 0.0001; fold change >1.5; gene rank: top 10%). For *CAMSAP1* expression in LIHC, GSE45267 dataset was also used. For CAMSAP1 protein expression in LIHC, immunohistochemical (IHC) images from the Human Protein Atlas portal (HPA, https://www.proteinatlas.org/) were used and the average optical density (AOD) of CAMSAP1 staining was measured by ImageJ software (NIH). For miRNA and lncRNA expression analyses, datasets from TCGA and/or GTEx were used. All these analyses were conducted with unpaired Wilcoxon rank-sum test and represented as histograms or violin plots.

### Genetic alteration and methylation level analysis

The cBioPortal (http://www.cbioportal.org/) was used to analyze genetic alteration (TCGA, Pan-Cancer Atlas) and DNA methylation (TCGA, Firehose) of *CAMSAP1*. Correlation plots of *CAMSAP1* expression with copy-number alterations (CNAs) and DNA methylation were downloaded directly from cBioPortal and displayed as heatmaps. Histograms representing *CAMSAP1* promoter methylation levels in normal tissue and primary tumor were downloaded from the UALCAN portal (http://ualcan.path.uab.edu/index.html). The beta value 0.5–0.7 was considered hyper-methylation, and 0.25–0.3 was hypo-methylation.

### Prediction of *CAMSAP1*-associated miRNAs and long non-coding ribonucleic acids

The Encyclopedia of RNA Interactomes portal (ENCORI, https://rna.sysu.edu.cn/encori/index.php) was used to analyze *CAMSAP1*-miRNA, lncRNA-miRNA, and lncRNA-*CAMSAP1* relationships in several cancers (Li et al., 2014). Firstly, seven target-predicting programs were used to predict the correlation between *CAMSAP1* and miRNA (parameter setting: assembly, hg38; CLIP-Data ≥ 5; pan-Cancer ≥ 1; programNum ≥ 2; target, *CAMSAP1*). Then, miRanda was used to predict the correlation between miRNA and lncRNA (parameter setting: assembly, hg38; miRNA: hsa-miR-101–3p; CLIP-Data ≥ 5; pan-Cancer ≥ 1) (Hoadley et al., 2014). All these correlation analyses were conducted online, and the resulting correlation plots were downloaded and displayed as lollipops.

### Diagnostic and survival analysis of *CAMSAP1*, miRNA, and lncRNA

The diagnostic values of *CAMSAP1*, miRNA and lncRNA were estimated by the receiver operating characteristic (ROC) curve using the pROC R package (Version 1.17.0.1, https://cran.r-project.org/web/packages/pROC/index.html). The area under the curve (AUC) > 0.9 was considered to represent high diagnostic value, whereas 0.7–0.9 was median, and 0.5–0.7 was low.

Survival analyses of *CAMSAP1*-upregulated and -downregulated cancers, including overall survival (OS), disease-specific survival (DSS), and progression-free interval (PFI), were conducted by Kaplan–Meier (KM) analyses and represented by a forest plot. For LIHC, patients were divided according to the expression of *CAMSAP1*, miRNAs, and lncRNAs, and KM analyses were used to evaluate the prognostic value. Survivin R (Version 3.2–10, https://cran.r-project.org/web/packages/survivalAnalysis/index.html) and survminer R package (Version 0.4.9, https://cran.r-project.org/web/packages/survminer/index.html) were used for statistical analyses and visualization.

### Correlation analyses between *CAMSAP1* expression and clinical features

The correlation between *CAMSAP1* expression and clinical features in LIHC from TCGA database was analyzed as a baseline datasheet using the Chi-square test or Fisher’s exact test. In addition, an unpaired Wilcoxon rank-sum test and logistic regression analysis were performed to product violin plots and forest plots, respectively.

### Clinical statistical analysis on prognosis, model construction, and evaluation

Univariate and multivariate Cox regression analyses were used to compare the effects of *CAMSAP1* expression and other clinical characteristics on survival rate. Bars represent 95% confidence intervals (CI) of hazard ratio (HR). Survivin R package was used for survival data statistics and represented by forest plots.

Based on multivariate Cox regression, nomogram was designed using the rms R package (Version 6.2–0, https://cran.r-project.org/web/packages/rms/index.html) and survival R package. Calibration curves and concordance index (C-index) were used to compare the accuracy of the nomogram model. The C-index > 0.9 indicated high accuracy, 0.7–0.9 indicated median, and 0.5–0.7 indicated low (Liu et al., 2018).

### Functional enrichment analysis of *CAMSAP1*-related differentially expressed genes

*CAMSAP1*-associated DEGs in LIHC were identified using the limma R package (Version 3.40.2, http://bioconductor.org/packages/3.9/bioc/src/contrib/Archive/limma/), and presented by a volcano plot (Love et al., 2014). The correlation between *CAMSAP1* and the top 20 DEGs was assessed through a heatmap. Functional enrichment analysis of these DEGs was conducted by Gene Ontology (GO) and Gene Set Enrichment Analysis (GSEA). GO analysis is a common method for annotating functional genes, especially cellular component (CC), molecular function (MF), and biological pathway (BP), and represented by bubble plots. GSEA was conducted to detect phenotypes and signaling pathways. Statistical analysis and graphical charting of GO and GSEA data were performed using the clusterProfiler R package (Version 3.14.3, http://bioconductor.riken.jp/packages/3.10/bioc/html/clusterProfiler.html) (Yu et al., 2012; Love et al., 2014).

### Immune infiltration analysis

Tumor Immune Estimation Resource 2 (TIMER2, http://timer.cistrome.org) is a comprehensive resource for systematic analysis of immune infiltrates across diverse cancer types. It was used to analyze the correlation between *CAMSAP1* expression and immune cells, immune cell markers, immune checkpoints, and cancer-associated fibroblasts (CAFs) in LIHC (Li T. et al., 2020; Wu et al., 2020; Yi et al., 2020). TCGA datasets were also used to evaluate the spearman correlation between *CAMSAP1* expression and immune cell markers, immune checkpoints, tumor mutation burden (TMB), and microsatellite instability (MSI) in LIHC, and represented by scatter plots or heatmaps (Bonneville et al., 2017; Thorsson et al., 2018).

Tumor Immune Dysfunction and Exclusion (TIDE, http://tide.dfci.harvard.edu/) module uses a set of markers to evaluate two different tumor immune escape mechanisms, including tumor-infiltrating cytotoxic T lymphocyte (CTL) dysfunction and rejection by immunosuppressive factors including immune checkpoint genes. High TIDE score indicated poor efficacy and short survival after ICB treatment.

## Results

### Pan-cancer expression of *CAMSAP1* is associated with genetic alteration and deoxyribonucleic acid methylation

**FIGURE 1:**
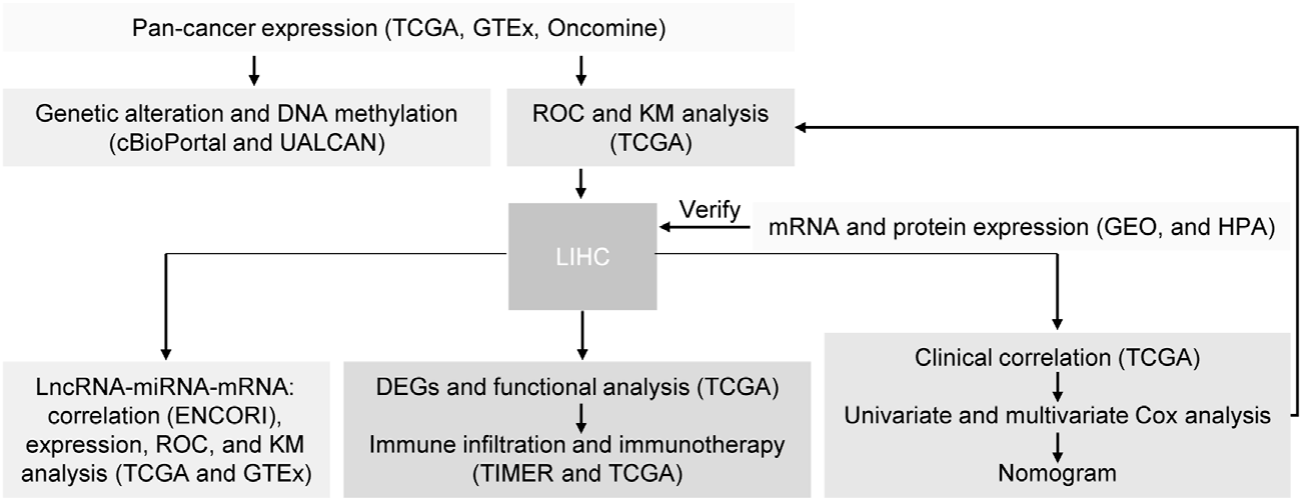
Schematic pipeline of data collection and analyses.

**FIGURE 2:**
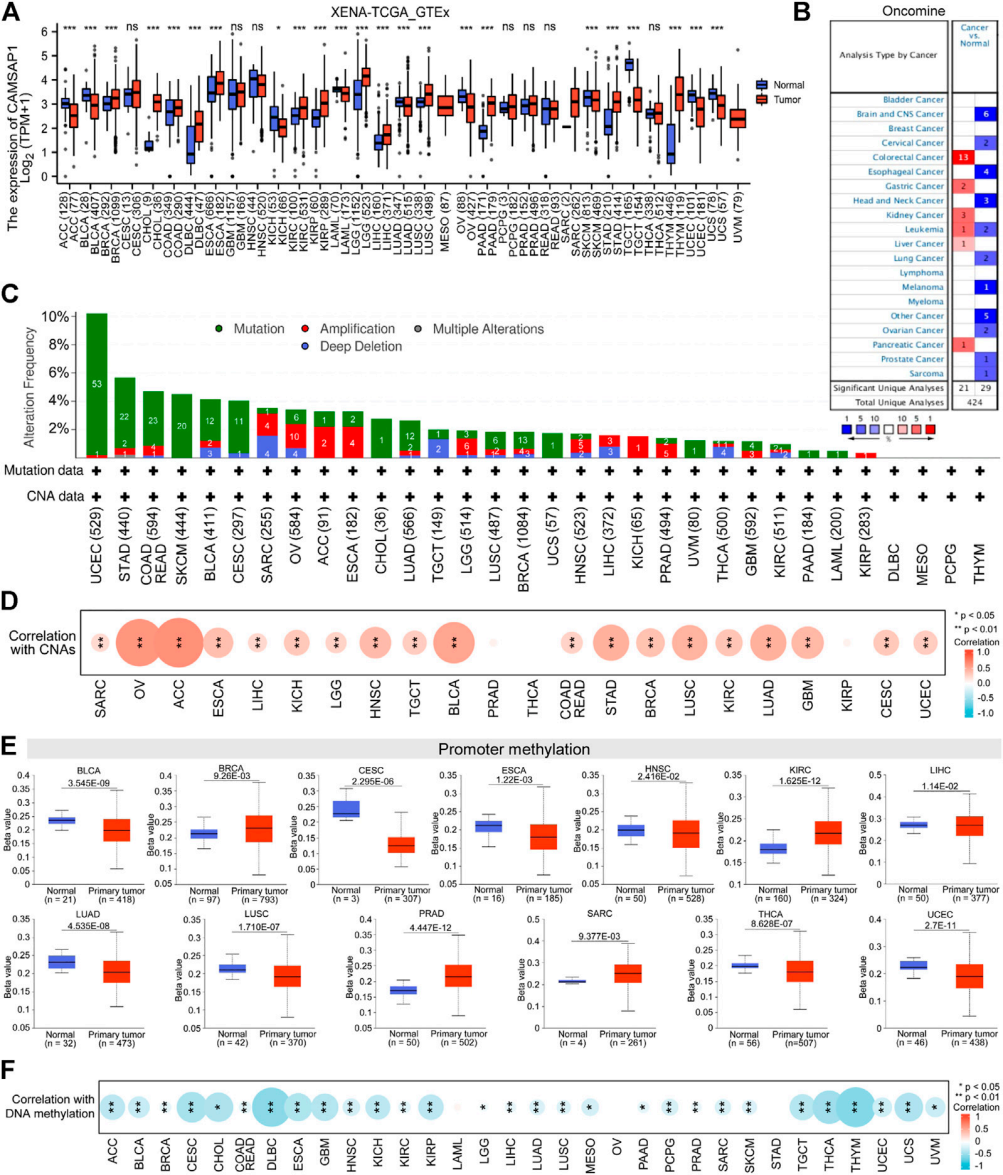
Pan-cancer expression of *CAMSAP1* is associated with genetic alteration and DNA methylation. **(A)** The expression of *CAMSAP1* in normal versus 33 types of tumor tissues from UCSC Xena database. ns: *p* ≥ 0.05, *p < 0.05, **p < 0.01, ***p < 0.001. ACC, adrenocortical carcinoma; BLCA, bladder urothelial carcinoma; BRCA, breast infiltrating carcinoma; CESC, cervical squamous cell carcinoma and endocervical adenocarcinoma; CHOL, cholangiocarcinoma; COAD, colon adenocarcinoma; DLBC, diffuse large B cell lymphoma; ESCA, esophageal carcinoma; GBM, glioblastoma multiforme; HNSC, head and neck squamous cell carcinoma, KICH, kidney chromophobe; KIRC, kidney renal clear cell carcinoma; KIRP, kidney renal papillary cell carcinoma; LAML, acute myeloid leukemia; LGG, brain low-grade glioma; LIHC, liver hepatocellular carcinoma; LUAD, lung adenocarcinoma; LUSC, lung squamous cell carcinoma; MESO, mesothelioma; OV, ovarian serous cystadenocarcinoma; PAAD, pancreatic adenocarcinoma; PCPG, pheochromocytoma and paraganglioma; PRAD, prostate adenocarcinoma; READ, rectum adenocarcinoma; SARC, sarcoma; SKCM, skin cutaneous melanoma; STAD, stomach adenocarcinoma; TGCT, testicular germ cell tumors; THCA, thyroid carcinoma; THYM, thymoma; UCEC, uterine corpus endometrial carcinoma; UCS, uterine carcinosarcoma; UVM, uveal melanoma. **(B)** The expression of *CAMSAP1* from the Oncomine portal. Red represented high expression in cancer, blue represented low expression in cancer, and the number in the table represented datasets that meet the threshold. The threshold was *p* < 0.0001, fold change >1.5 and gene rank = 10%. **(C,D)** *CAMSAP1* genetic variation **(C)** and its correlation with CNAs **(D)** in pan-cancer from cBioPortal. **(E)** *CAMSAP1* promoter methylation in normal and primary tumor from UALCAN portal. **(F)** Correlation between *CAMSAP1* and DNA methylation from cBioPortal. *p < 0.05, **p < 0.01.

To explore the possible mechanism of *CAMSAP1* in tumorigenesis, we conducted a series of data collection and analyses (Figure 1). First, we compared its expression in 33 types of human cancer and normal samples using datasets from the UCSC Xena database. *CAMSAP1* expression was found to be upregulated in breast infiltrating carcinoma (BRCA), cholangiocarcinoma (CHOL), colon adenocarcinoma (COAD), diffuse large B cell lymphoma (DLBC), esophageal carcinoma (ESCA), kidney renal clear cell carcinoma (KIRC), kidney renal papillary cell carcinoma (KIRP), low-grade glioma (LGG), LIHC, lung squamous cell carcinoma (LUSC), pancreatic adenocarcinoma (PAAD), stomach adenocarcinoma (STAD), and thymoma (THYM). In contrast, its expression was significantly downregulated in adrenocortical carcinoma (ACC), bladder urothelial carcinoma (BLCA), kidney chromophobe (KICH), bladder urothelial carcinoma (LAML), lung adenocarcinoma (LUAD), ovarian serous cystadenocarcinoma (OV), skin melanoma (SKCM), testicular germ cell tumors (TGCT), uterine corpus endometrial carcinoma (UCEC), and uterine carcinosarcoma (UCS) (*p* < 0.05). However, there was no significant difference in *CAMSAP1* expression in cervical squamous cell carcinoma and endocervical adenocarcinoma (CESC), glioblastoma multiforme (GBM), head and neck squamous cell carcinoma (HNSC), pheochromocytoma and paraganglioma (PCPG), prostate cancer (PRAD), rectum adenocarcinoma (READ), and thyroid cancer (THCA) (Figure 2A). Next, we used the Oncomine database to verify *CAMSAP1* expression and found that the expression was significantly increased in colorectal, gastric, kidney, liver, and pancreatic cancers. In contrast, its expression was decreased in brain and central nervous system, cervical, esophageal, head and neck, lung, ovarian, and prostate cancers, as well as in melanoma and sarcoma (Figure 2B).

The susceptibility of genetic variation to cancer is well known. Therefore, we first used cBioPortal (TCGA, Pan-Cancer Atlas) to study the relationship between *CAMSAP1* expression and genetic variation. *CAMSAP1* genetic variation included amino acid mutation and CNAs, among these 32 cancer types (COAD and READ are merged into COADREAD), 25 of them contained *CAMSAP1* mutation, and 22 had CNAs (Figure 2C). UCEC and STAD had the highest alteration frequency (>5%), while DLBC, mesothelioma (MESO), PCPG, and THYM had no alteration. CNAs are the genetic variation most associated with *CAMSAP1* expression (Lee et al., 2008), and most of the 22 cancers showed positive correlation with its expression, especially ACC (*r* = 0.64, *p* = 4.03e-10), OV (*r* = 0.61, *p* = 9.43e-32), and BLCA (*r* = 0.53, *p* = 6.56e-31) (Figure 2D; Supplementary Table S1). DNA methylation directly affects mRNA expression (Men et al., 2017; Skvortsova et al., 2019). As one of the regulation mechanisms, *CAMSAP1* expression and promoter methylation level were not completely uniform. The methylation level was decreased in BLCA, CESC, ESCA, HNSC, LIHC, LUAD, LUSC, THCA, and UCEC tissues compared to normal tissues according to the UALCAN portal; meanwhile, it was greatly increased in BRCA, KIRC, PRAD, and SARC (*p* < 0.05) (Figure 2E). However, no significant difference was observed in other cancers (Supplementary Figure S1). Next, we investigated the correlation between *CAMSAP1* expression and DNA methylation from cBioPortal (TCGA, firehose). DNA methylation was negatively correlated with *CAMSAP1* expression, especially for THYM (*r* = −0.58, *p* = 4.94e-12), DLBC (*r* = −0.57, *p* = 2.628e-5), and THCA (*r* = −0.48, *p* = 4.19e-31) (Figure 2F; Supplementary Table S2).

Combined with UCSC Xena and Oncomine databases, CHOL, COAD, KIRC, KIRP, LIHC, PAAD, and STAD were chosen for *CAMSAP1* upregulation studies, whereas LUAD, OV, and SKCM were selected for downregulation research. Taken together, these results suggest that *CAMSAP1* expression may play an important regulatory role in the carcinogenesis of at least 10 cancer types.

### Differentially expressed *CAMSAP1* serves as a potential diagnostic and prognostic biomarker in some cancers

**FIGURE 3:**
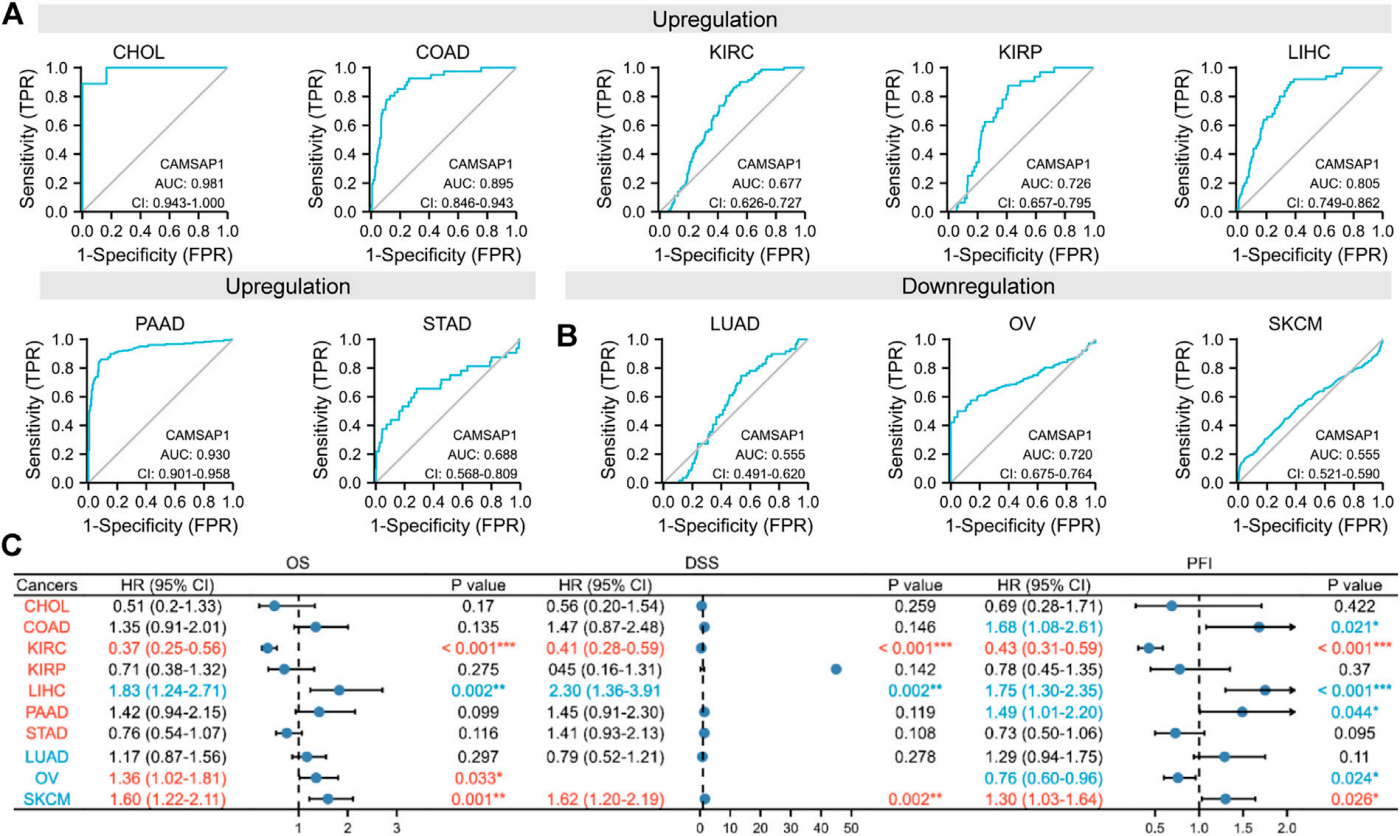
Differentially expressed *CAMSAP1* serves as a potential diagnostic and prognostic biomarker in some cancers. **(A,B)** ROC analyses of *CAMSAP1*. **(C)** A forest plot of survival analyses with OS, DSS, and PFI. *p < 0.05, **p < 0.01, ***p < 0.001.

To confirm the suggestion, we evaluated the diagnostic value and survival effect of *CAMSAP1* in cancers. ROC analyses indicated that *CAMSAP1* was a diagnostic molecule with significantly high or median values in CHOL (AUC = 0.981), COAD (0.895), KIRP (0.726), LIHC (0.805), PAAD (0.930), and OV (0.720), whereas low values in KIRC (0.677), STAD (0.688), LUAD (0.555), and SKCM (0.555) (Figures 3A,B).

Prognostic analyses were then performed (Figure 3C; Supplementary Figure S2). In the *CAMSAP1*-upregulated groups, LIHC patients had poor prognosis, including OS (HR = 1.83, 95% CI = 1.24–2.71, *p* = 0.002), DSS (HR = 2.30, 95% CI = 1.36–3.91, *p* = 0.002), and PFI (HR = 1.75, 95% CI = 1.30–2.35, *p* < 0.001); meanwhile, COAD (HR = 1.68, 95% CI = 1.08–2.61, *p* = 0.021) and PAAD (HR = 1.49, 95% CI = 1.01–2.20, *p* = 0.044) patients only had poor PFI. However, KIRC patients exhibited favorable prognosis for OS (HR = 0.37, 95% CI = 0.25–0.56, *p* < 0.001), DSS (HR = 0.41, 95% CI = 0.28–0.59, *p* < 0.001), and PFI (HR = 0.43, 95% CI = 0.31–0.59, *p* < 0.001). Surprisingly, in the *CAMSAP1*-downregulated groups, OS (HR = 1.36, 95% CI = 1.02–1.81, *p* = 0.033) for OV patients, and OS (HR = 1.60, 95% CI = 1.22–2.11, *p* = 0.001), DSS (HR = 1.62, 95% CI = 1.20–2.19, *p* = 0.002), and PFI (HR = 1.30, 95% CI = 1.03–1.64, *p* = 0.026) for SKAM patients were increased significantly. Due to the low diagnostic value in KIRC and SKCM, *CAMSAP1* serves as a novel diagnostic and prognostic biomarker in LIHC.

### Overexpressed *CAMSAP1* leads to adverse clinical outcomes in advanced liver hepatocellular carcinoma

**FIGURE 4:**
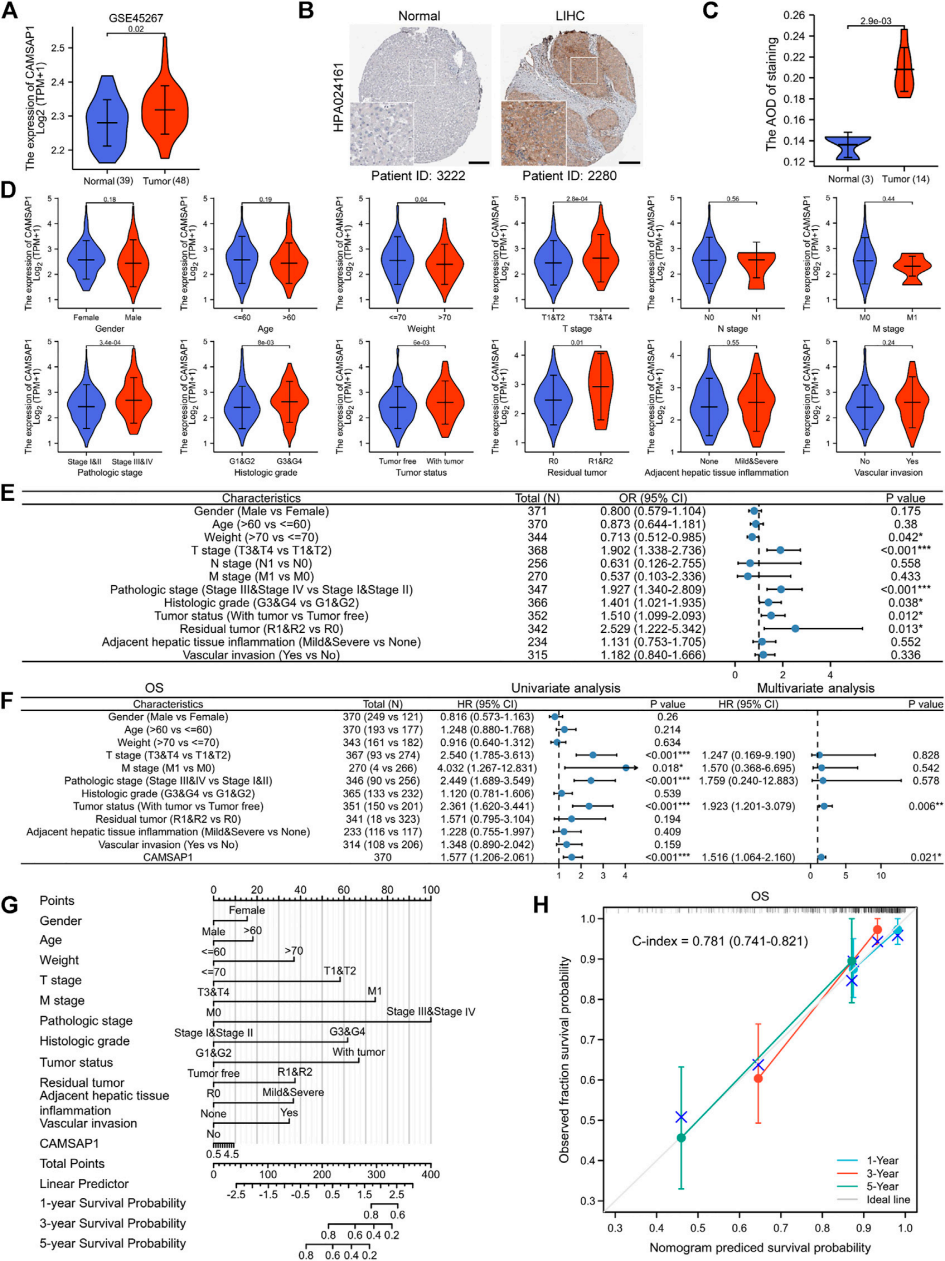
Overexpressed *CAMSAP1* leads to adverse clinical outcomes in advanced LIHC. **(A)** The expression of *CAMSAP1* in LIHC from the GEO database. **(B)** IHC images of CAMSAP1 proteins with antibody HPA024161 in normal and LIHC tissues from the HPA database. Scare bar, 200 μm. **(C)** Statistical analysis of CAMSAP1 IHC intensity. **(D)** Violin plots of correlation between *CAMSAP1* expression and clinical features. **(E)** A forest plot of univariate Logistic regression analysis of the correlation between *CAMSAP1* expression and clinical features. **(F)** A forest plot of univariate and multivariate Cox regression analyses with OS. *p < 0.05, **p < 0.01, ***p < 0.001. **(G)** A nomogram prediction model with 1-, 3-, and 5-years OS. **(H)** Nomogram calibration analysis with OS.

Dataset from GEO further confirmed the high expression of *CAMSAP1* in LIHC (GSE45267, *p* = 0.02) (Figure 4A). IHC staining from the HPA portal also showed prominent CAMSAP1 antibody staining in LIHC tissue (*p* = 2.9e-03) (Figures 4B,C; Supplementary Figure S3). Based on these findings, we confirmed that *CAMSAP1* mRNA and protein are significantly upregulated in LIHC.

Subsequently, clinical data from 371 LIHC patients were obtained from TCGA database to evaluate the relationship between *CAMSAP1* expression and clinical features (Table 1). The cohort comprised 121 females (32.6%) and 250 males (67.4%) with an average age and weight of 60 years and 70 kg, respectively. Results showed that *CAMSAP1* expression was significantly correlated with the weight (*p* = 0.04), T stage (2.8e-04), pathologic stage (3.4e-04), histologic grade (8e-03), tumor status (6e-03), and residual tumor (0.01) (Figure 4D). Univariate logistic regression analysis further demonstrated that *CAMSAP1* expression was a categorical dependent variable associated with clinical features of adverse prognosis. *CAMSAP1* expression was positively correlated with weight (odds ratio (OR) = 0.713, 95% CI = 0.512–0.985, *p* = 0.042), T stage (OR = 1.902, 95% CI = 1.338–2.736, *p* < 0.001), pathologic stage (OR = 1.927, 95% CI = 1.340–2.809, *p* < 0.001), histologic grade (OR = 1.401, 95% CI = 1.021–1.935, *p* = 0.038), tumor status (OR = 1.510, 95% CI = 1.099–2.093, *p* = 0.012), and residual tumor (OR = 2.529, 95% CI = 1.222–5.342, *p* = 0.013) (Figure 4E). These results suggest that high *CAMSAP1* expression is more likely to be observed in LIHC patients with more advanced stage and grade.

**TABLE 1:** Correlation between *CAMSAP1* expression and clinical variables in LIHC based on TCGA database.

Characteristic	Low expression	High expression	*p*
n	185	186	
Gender, n (%)			0.130
Female	53 (14.3%)	68 (18.3%)	
Male	132 (35.6%)	118 (31.8%)	
Race, n (%)			0.498
Asian	80 (22.3%)	78 (21.7%)	
Black or African American	10 (2.8%)	7 (1.9%)	
White	85 (23.7)	99 (27.6%)	
Age, n (%)			0.096
≤60	80 (21.6%)	97 (26.2%)	
>60	105 (28.4%)	88 (23.8%)	
Weight, n (%)			0.100
≤70	85 (24.7%)	97 (28.2%)	
>70	91 (26.5%)	71 (20.6%)	
T stage, n (%)			0.046^*^
T1	102 (27.7%)	79 (21.5%)	
T2	44 (12%)	50 (13.6%)	
T3	31 (8.4%)	49 (13.3%)	
T4	5 (1.4%)	8 (2.2%)	
N stage, n (%)			1.000
N0	117 (45.7%)	135 (52.7%)	
N1	2 (0.8%)	2 (0.8%)	
M stage, n (%)			0.361
M0	129 (47.8%)	137 (50.7%)	
M1	3 (1.1%)	1 (0.4%)	
Pathologic stage, n (%)			0.008^**^
Stage I	95 (27.4%)	76 (21.9%)	
Stage II	41 (11.8%)	45 (13%)	
Stage III	30 (8.6%)	55 (15.9%)	
Stage IV	4 (1.2%)	1 (0.3%)	
Histologic grade, n (%)			0.003^**^
G1	36 (9.8%)	19 (5.2%)	
G2	95 (26%)	82 (22.4%)	
G3	46 (12.6%)	76 (20.8%)	
G4	5 (1.4%)	7 (1.9%)	
Tumor status, n (%)			0.018^*^
Tumor free	112 (31.8%)	89 (25.3%)	
With tumor	64 (18.2%)	87 (24.7%)	
Residual tumor, n (%)			0.025^*^
R0	168 (49.1%)	156 (45.6%)	
R1	4 (1.2%)	13 (3.8%)	
R2	1 (0.3%)	0 (0%)	
Adjacent hepatic tissue inflammation, n (%)			0.17
None	67 (28.6%)	50 (21.4%)	
Mild	44 (18.8%)	55 (23.5%)	
Severe	9 (3.8%)	9 (3.8%)	
Vascular invasion, n (%)			0.024^*^
No	114 (36.2%)	92 (29.2%)	
Yes	45 (14.3%)	64 (20.3%)	

*p < 0.05; **p < 0.01.

To further confirm the relationship between *CAMSAP1* expression and poor prognostic outcome in LIHC, we performed additional univariate Cox regression analyses. Overall, high *CAMSAP1* expression was significantly correlated with poor OS (HR = 1.577, 95% CI = 1.206–2.061, *p* < 0.001) (Figure 4F, left) and DSS (HR = 1.495, 95% CI = 1.051–2.126, *p* = 0.025) (Supplementary Figure S4A, left) as well as poor PFI (HR = 1.554, 95% CI = 1.234–1.957, *p* < 0.001) (Supplementary Figure S5A, left). To identify factors related to survival, multivariate Cox regression analysis was performed. *CAMSAP1* expression remained an independent factor associated with OS (HR = 1.516, 95% CI = 1.064–2.16, *p* = 0.021) (Figure 4F, right) and PFI (HR = 1.440, 95% CI = 1.035–2.004, *p* = 0.031) (Supplementary Figure S5A, right). However, *CAMSAP1* expression had no association with DSS (HR = 1.422, 95% CI = 0.892–2.267, *p* = 0.139) (Supplementary Figure S4A, right). Based on multivariate Cox regression analyses, a nomogram prediction model was established using *CAMSAP1* expression and other independent prognostic factors. Probabilities of 1-, 3-, and 5-years survival were predicted, including OS (Figure 4G), DSS (Supplementary Figure S4B), and PFI (Supplementary Figure S5B). To verify the validity of the predictive model, we performed calibration analysis on the nomogram. The results confirmed that the C-index of OS was 0.781 (0.741–0.821) (Figure 4H) and that of DSS was 0.813 (0.78–0.846) (Supplementary Figure S4C), indicating a median accuracy. For the PFI model, C-index was 0.665 (0.633–0.697) (Supplementary Figure S5C), indicating relatively low accuracy.

### The AC145207.5/LINC01748-miR-101–3p axis is specifically responsible for *CAMSAP1* overexpression in liver hepatocellular carcinoma

**FIGURE 5:**
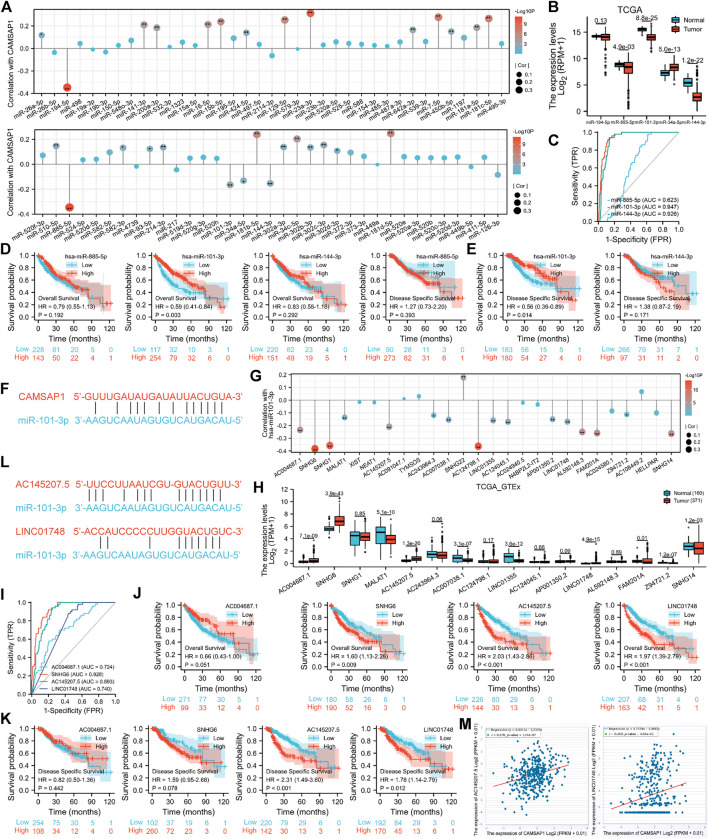
The AC145207.5/LINC01748-miR-101–3p axis is specifically responsible for *CAMSAP1* overexpression in LIHC. **(A,G)** Lollipop plots of spearman correlation between *CAMSAP1* and predicted miRNAs **(A)**, miR-101–3p, and predicted lncRNAs **(G)** from ENCORI portal. *p < 0.05, **p < 0.01. **(B,H)** The expression of negatively correlated miRNAs of *CAMSAP1* **(B)** and negatively correlated lncRNAs of miR-101–3p **(H)** in normal and LIHC tissues from TCGA and TCGA plus GTEx database. *p < 0.05, **p < 0.01, ***p < 0.001. **(C,I)** ROC analyses of negatively correlated miRNAs **(C)** and lncRNAs **(H)**. **(D,E,J,K)** KM analyses for OS and DSS of negatively correlated miRNAs **(D,E)** and lncRNAs **(J,K)**. **(F,L)** The pairing sequence of *CAMSAP1*-miR-101–3p **(F)**, miR-101-3p-AC145207.5 and miR-101-3p-LINC01748 **(L)**. **(M)** Scatter plots of correlation between *CAMSAP1* and AC145207.5 and LINC01748. *p* < 0.05 indicated a significant difference.

In addition to genetic alteration and DNA methylation regulation in pan-cancer, we introduced ncRNAs well-known for their role in regulating gene expression to further explore the regulatory mechanism of *CAMSAP1* overexpression in LIHC (Anastasiadou et al., 2018). Firstly, the ENCORI portal predicted upstream miRNAs that could bind to *CAMSAP1* in several cancers and a total of 74 miRNAs were found in pan-cancer (Supplementary Table S3). Typically, miRNA binds and attenuates target mRNA expression, and there is a negative correlation between them. Therefore, we further searched for miRNAs negatively correlated with *CAMSAP1*. *CAMSAP1* expression was significantly negatively correlated with five of them, including miR-194–5p, miR-885–5p, miR-101–3p, miR-34a-5p, miR-144–3p, whereas positively correlated with 24 miRNAs (Figure 5A). Among these five negatively correlated miRNAs, miR-885–5p (*p* = 4.9e-03), miR-101–3p (8.8e-25), and miR-144–3p (1.2e-22) were markedly downregulated in LIHC from TCGA database (Figure 5B). ROC analysis indicated that miR-101–3p (AUC = 0.947) and miR-144–3p (0.926) had significantly high diagnostic value whereas that of miR-885–5p was low (0.623) (Figure 5C). In addition, KM analysis showed that upregulated miR-101–3p had favorable prognosis potential for both OS (*p* = 0.003) (Figure 5D) and DSS (0.014) (Figure 5E). The pairing sequence of miR-101–3p and *CAMSAP1* was displayed (Figure 5F). In conclusion, miR-101–3p is the most promising miRNA for regulating *CAMSAP1* in LIHC.

Next, we used the ENCORI to predict the upstream lncRNAs of miR-101–3p in pan-cancers and a total of 26 predicted lncRNAs were screened out (Supplementary Table S4). Based on the regulatory mechanism of lncRNA-miRNA, there should be a negative correlation. miR-101–3p was significantly negatively and positively correlated with 16 and one lncRNAs, respectively (Figure 5G). Among the 16 negatively correlated lncRNAs, AC004687.1 (*p* = 7.1e-09), SNHG6 (3.9e-43), AC145207.5 (1.3e-20), and LINC01748 (4.9e-15) were significantly upregulated in LIHC from TCGA plus GTEx databases (Figure 5H). Subsequently, ROC analysis indicated that SNHG6 (AUC = 0.928) had a significantly high diagnostic value; AC145207.5 (0.893), LINC01748 (0.740), and AC004687.1 (0.724) had median values (Figure 5I). KM survival analysis showed that only LIHC patients with upregulated SNHG6 (*p* = 0.009), AC145207.5 (<0.001) and LINC01748 (<0.001) showed poor OS (Figure 5J). Furthermore, upregulated AC145207.5 (<0.001), and LINC01748 (0.012) were also associated with poor DSS (Figure 5K). The pairing sequences of miR-101-3p-AC145207.5 and miR-101-3p-LINC01748 were displayed (Figure 5L). Collectively, AC145207.5 and LINC01748 are the potential regulatory lncRNAs of miR-101–3p in LIHC.

According to the competing endogenous RNA (ceRNA) hypothesis (Tay et al., 2014; Qi et al., 2015), lncRNA may boost mRNA expression by sharing miRNA binding. Therefore, lncRNA should correlate negatively with miRNA, but positively with mRNA. The correlation between the two lncRNAs and *CAMSAP1* were also detected, with AC145207.5 (r = 0.270, *p* = 1.14e-07) and LINC01748 (r = 0.209, *p* = 4.45e-05) being positively correlated with *CAMSAP1* (Figure 5M). Together, we confirmed that the AC145207.5/LINC01748-miR-101–3p constitutes an upstream ncRNA axis that regulates *CAMSAP1* expression in LIHC.

### *CAMSAP1*-related differentially expressed genes are enriched in immune-associated pathways

**FIGURE 6:**
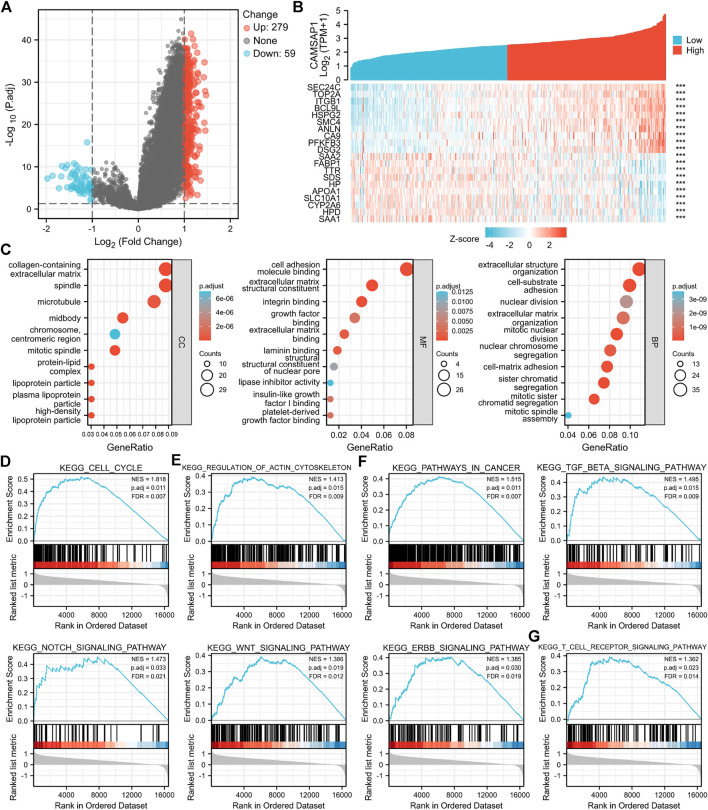
*CAMSAP1*-related DEGs are enriched in immune-associated pathways. **(A)** A volcano plot of *CAMSAP1*-related DEGs. **(B)** A heatmap of correlation between *CAMSAP1* and the top 20 DEGs. ***p < 0.001. **(C)** Bubble plots from GO enrichment. **(D–G)** Enrichment plots from GSEA. *p* < 0.05 and FDR <0.05 were considered the meaningful pathway.

Based on the different expression of *CAMSAP1* between normal and LIHC tissues, LIHC patients were divided into high- and low-*CAMSAP1* expression groups, and the mRNA profile of the two groups was compared. A total of 338 DEGs were found to be statistically significant between the two groups, including 279 upregulated and 59 downregulated genes (adjusted *p* < 0.05 and |log_2_FC| > 1) (Figure 6A). The correlation between *CAMSAP1* and the top 20 DEGs are illustrated in a heatmap (Figure 6B; Supplementary Table S5).

To further predict the functional enrichment of *CAMSAP1* interactive genes in LIHC, GO and GSEA enrichment analyses were performed. Overall, *CAMSAP1*-related DEGs were enriched in 1) CCs: the collagen-containing extracellular matrix, spindle, and microtubule; 2) MFs: cell adhesion molecule binding, extracellular matrix structural constituent, and integrin binding; and 3) BPs: extracellular structure organization, cell-substrate adhesion, and nuclear division (Figure 6C; Supplementary Table S6). GSEA enrichment was performed to identify *CAMSAP1* expression-related signaling pathways. Results suggested that genes were significantly enriched in the cell cycle (Figure 6D), regulation of actin cytoskeleton (Figure 6E), cancer-related pathways (Pathways in cancer, TGF-beta signaling pathway, Notch signaling pathway, Wnt signaling pathway, Erbb signaling pathway) (Figure 6F), and immune system (T cell receptor signaling pathway) (Figure 6G), implying that *CAMSAP1*-related genes may also be involved in immune regulation (Supplementary Table S7).

### *CAMSAP1*-associated liver hepatocellular carcinoma is infiltrated in the suppressed immune microenvironment

**FIGURE 7:**
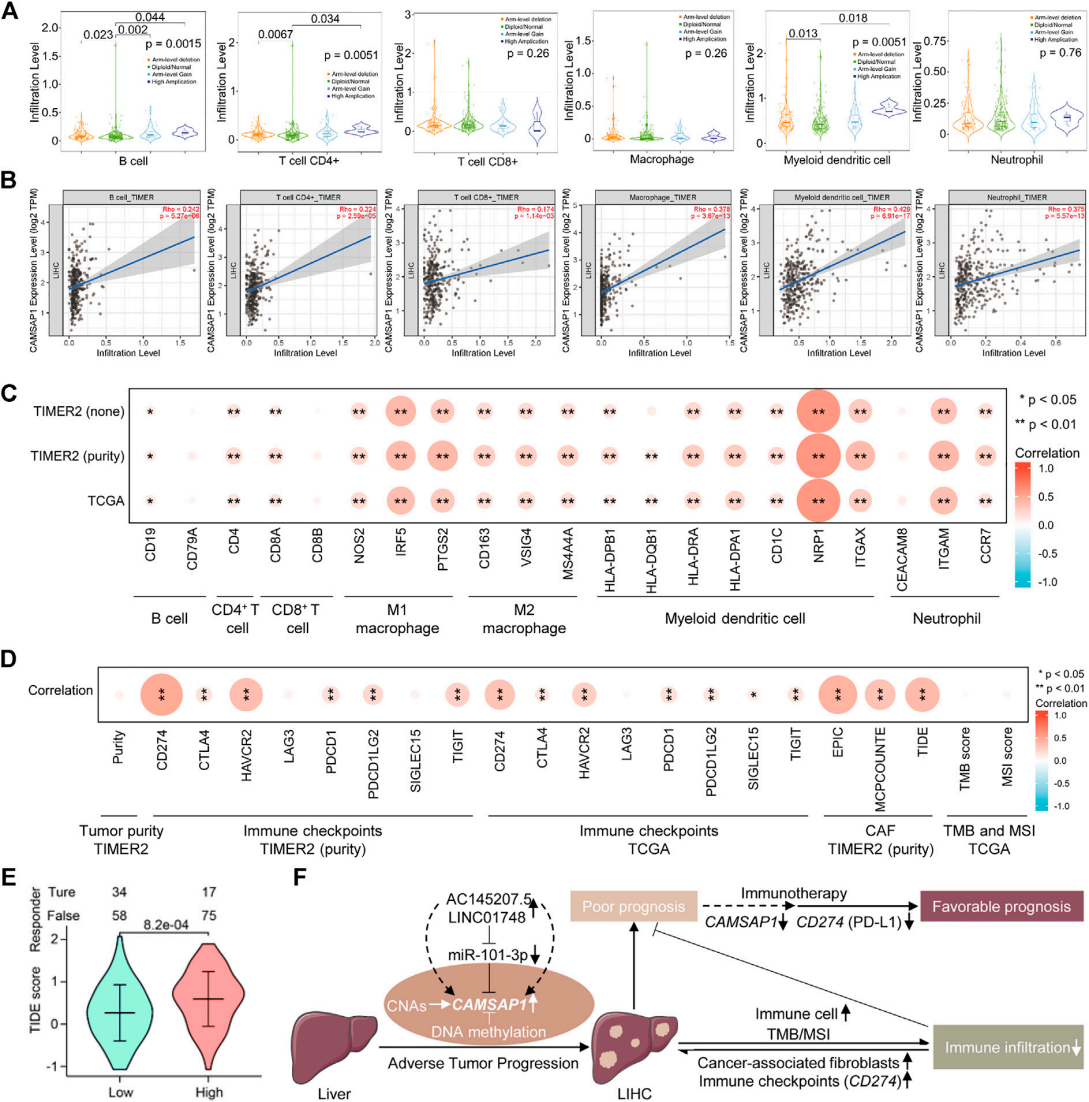
*CAMSAP1*-associated LIHC is infiltrated in the suppressed immune microenvironment. **(A)** The infiltration level of six major immune cells under different copy numbers of *CAMSAP1* using TIMER2. **(B)** Scatter plots of correlation between *CAMSAP1* and various immune cells from TIMER2. *p* < 0.05 indicated a significant difference. **(C,D)** Heatmaps of correlation between *CAMSAP1* and immune cell markers **(C)** and immune regulated genes **(D)** from TIMER2 and TCGA. *p < 0.05, **p < 0.01. **(E)** A violin plot of *CAMSAP1*-associated ICB treatment effect between high and low *CAMSAP1* expression by TIDE algorithm. *p* < 0.05 indicated a significant difference. **(F)** Possible model underlying the AC145207.5/LINC01748-miR-101-3p-*CAMSAP1* axis works on immune infiltration to improve the prognosis of LIHC.

The tumor immune microenvironment is complex, generally being positively regulated by immune cell infiltration and negatively regulated by immune checkpoints, CAFs, and other factors, thus determining the tumor prognosis. The GSEA enrichment of *CAMSAP1*-associated genes implied that they could function in immune regulation. Therefore, we investigated whether *CAMSAP1* expression in LIHC was associated with immune infiltration using the TIMER2 portal. From *CAMSAP1* arm-level deletion to high amplification, B cell (*p* = 0.0015), CD4^+^ T cell (0.0051), and myeloid dendritic cell (0.0051) infiltration levels were increased (Figure 7A). *CAMSAP1* expression was positively correlated with all the immune cells analyzed, including B cells (Rho = 0.242, *p* = 5.27e-06), CD4^+^ T cells (Rho = 0.224, *p* = 2.59e-05), CD8^+^ T cells (Rho = 0.174, *p* = 1.14e-03), macrophages (Rho = 0.378, *p* = 3.67e-13), myeloid dendritic cells (Rho = 0.429, *p* = 6.91e-17), and neutrophils (Rho = 0.375, *p* = 5.57e-13) in LIHC using TIMER2 portal, especially for those of macrophages, myeloid dendritic cell, and neutrophil (Rho >0.3, *p* < 0.001) (Figure 7B). Next, we evaluated the relationship between *CAMSAP1* and various immune cell markers in LIHC using the TIMER2 and TCGA databases to further explore the role of *CAMSAP1* in immune infiltration. The expression of *CAMSAP1* was positively correlated with most of these genes, especially IRF5 and PTGS2 of M1 macrophages, NRP1 and ITGAX of myeloid dendritic cells, and ITGAM of neutrophils (Rho >0.3, *p* < 0.001) (Figure 7C; Supplementary Figure S6A; Supplementary Table S8). These findings support that *CAMSAP1* is positively correlated to immune cell infiltration, especially innate immune cells.

Based on the previous results, we also conducted a comprehensive study from immune suppression and promotion to further explore the regulation mechanism of *CAMSAP1*-related LIHC immune infiltration. Important immune checkpoint genes responsible for tumor immune escape include *CD274*, *CTLA4*, *HAVCR2*, *LAG3*, *PDCD1*, *PDCD1LG2*, *SIGLEC15*, and *TIGIT*. Considering the possible tumorigenic role of *CAMSAP1* in LIHC, we evaluated the correlation between *CAMSAP1* expression and these genes. *CAMSAP1* expression had no correlation with LIHC purity, *LAG3*, and *SIGLEC15*, whereas it was positively correlated with *CD274* (Rho = 0.486, *p* = 7.06e-22), *CTLA4* (Rho = 0.169, *p* = 1.58e-03), *HAVCR2* (Rho = 0.377, *p* = 4.49e-13), *PDCD1* (Rho = 0.186, *p* = 5.15e-4), *PDCD1LG2* (Rho = 0.221, *p* = 3.57e-05), and *TIGIT* (Rho = 0.265, *p* = 6.15e-07) in LIHC using the TIMER2, especially for *HAVCR2*, and *CD274* (Rho >0.3, *p* < 0.001). Correlation analysis using TCGA dataset also confirmed that *CAMSAP1* was positively correlated with *CD274* (*r* = 0.341, *p* < 0.001), *CTLA4* (*r* = 0.15, *p* = 0.004), *HAVCR2* (*r* = 0.27, *p* < 0.001), *PDCD1* (*r* = 0.177, *p* = 0.001), *PDCD1LG2* (*r* = 0.158, *p* = 0.002), *SIGLEC15* (*r* = 0.117, *p* = 0.024), *TIGIT* (*r* = 0.165, *p* = 0.001), especially *CD274* (*r* > 0.3, *p* < 0.001), whereas *LAG3* was not correlated with *CAMSAP1*. Previous studies have indicated that CAFs in the stroma regulate different tumor infiltrating immune cells and participate in immunosuppression. Therefore, we employed the EPIC, MCPCOUNTE, and TIDE algorithms using TIMER2 to investigate the correlation between CAFs and *CAMSAP1* expression. Results showed that *CAMSAP1* expression was positively correlated with CAFs infiltration in LIHC (Rho = 0.442, *p* = 5.86e-18; Rho = 0.346, *p* = 3.89e-11; Rho = 0.393, *p* = 3.72e-14) (Figure 7D; Supplementary Figure S6B; Supplementary Table S9).

TMB and MSI are two emerging biomarkers that promote immune cell infiltration and reflect patient response to immunotherapy. For tumor cells with a higher TMB score, the stronger antigenicity it has, the more tumor cells can be recognized by immune cells, thus promoting immune infiltration. Tumor cells with higher MSI scores indicate that somatic cells are over-mutated and the tumor expresses more neoantigens, thus promoting immune cell infiltration. Unfortunately, *CAMSAP1* expression was not correlated with TMB (*r* = −0.035, *p* = 0.541) or MSI (*r* = 0.056, *p* = 0.287) (Figure 7D; Supplementary Figures S6C,D; Supplementary Table S9).

Next, we used the TIDE algorithm to predict the ICB treatment effect. Results showed that compared with the high *CAMSAP1* expression group, the low group exhibited a lower TIDE score, indicating a better therapeutic outcome (Figure 7E; Supplementary Table S10). Collectively, these results demonstrated that in addition to immune cell infiltration, tumor immune escape mediated by immune checkpoints and CAFs is also involved in *CAMSAP1*-mediated LIHC progression and ICB treatment is effective in these patients.

## Discussion

The early stages of LIHC are typically asymptomatic or atypical; thus, elucidating the underlying molecular mechanisms of LIHC may contribute to the identification of interesting diagnostic and prognostic biomarkers or the development of viable therapeutic targets. This study conducted pan-cancer analyses of *CAMSAP1* expression, and demonstrated that *CAMSAP1* upregulation was associated with advanced tumor and unfavorable prognosis in LIHC.

It is well known that ncRNAs, comprising miRNAs, lncRNAs, and circular RNAs, communicate with each other and play a role in regulating gene expression (Anastasiadou et al., 2018). Therefore, ENCORI were used to investigate the upstream regulated miRNAs of *CAMSAP1*. Five miRNAs were found to be negatively correlated with *CAMSAP1*, and three of them were downregulated in LIHC. Previous studies have demonstrated that most miRNAs serve as tumor-suppressors in LIHC. For example, miR-885–5p inhibits the Warburg effect by suppressing hexokinase 2 in LIHC (Xu et al., 2019), miR-101–3p renders LIHC cells more sensitive to oxaliplatin by suppressing Beclin-1-mediated autophagy (Sun et al., 2019), and miR-144-3p-mediated *EIF4G2* dysregulation promotes the development of LIHC via the ERK pathway (Li et al., 2021). It was also shown that downregulation of miR-101–3p expression can promote the proliferation and migration of LIHC cells (Sheng et al., 2014). As for the *CAMSAP1* regulatory miRNA, it has been previously reported that the downregulation of *CAMSAP1* in primary human osteoblasts and laryngeal squamous cell carcinoma is caused by upregulation of miR-126 (Sun et al., 2014; Strassburg et al., 2017). Our study also found that miR-101–3p is the regulatory miRNA of *CAMSAP1* expression in LIHC. Based on the ceRNA hypothesis (Tay et al., 2014; Qi et al., 2015), potential lncRNA regulators of the miR-101-3p-*CAMSAP1* axis are carcinogenic in LIHC. Therefore, upstream lncRNAs were evaluated, and 16 possible candidates were identified. Expression, diagnosis, and survival analyses identified two lncRNAs (AC145207.5 and LINC01748) most likely to be upregulated. These two lncRNAs reportedly function as oncogenes in various malignant tumors or autoimmune disease. For example, AC145207.5 (RP11-498C9.15) plays a pivotal role in rheumatoid arthritis by modulating both miRNAs and gene expression (Dolcino et al., 2019). Moreover, LINC01748 regulates the miRNA-520a-5p/HMGA1 axis to exert carcinogenic effects in non-small cell lung cancer (Tan et al., 2022). Collectively, the AC145207.5/LINC01748-miR-101-3p-*CAMSAP1* axis has been identified as a potential regulatory pathway in LIHC.

The tumor microenvironment is primarily composed of tumor cells, immune cells, and other cells including tumor-associated fibroblasts, extracellular matrix, vascular system, and their interacting cytokines and chemokines, and plays an important role in tumor progression (Reinfeld et al., 2021). Previous studies have confirmed that immune infiltration can influence the efficacy and prognosis of chemotherapy, radiotherapy, or immunotherapy in patients with cancer (Thorsson et al., 2018; Hiam-Galvez et al., 2021; Ruf et al., 2021). Although significant advances have been made in the immunotherapy field, challenges persist that impede its effectiveness. Therefore, identifying novel targets and biomarkers is the key to further improving the efficacy of immunotherapy. Meanwhile, a comprehensive understanding of immune infiltration is particularly important for patients with cancer to ensure that the most efficient and individualized immunotherapy strategy is selected. Our research revealed that *CAMSAP1*-related genes were enriched in cancer and the immune system. Our work further indicated that *CAMSAP1* was positively correlated with the major types of immune cells, suggesting that tumor immune infiltration may exert an antitumor effect in *CAMSAP1*-mediated LIHC. However, the effectiveness of immunotherapy depends not only on immune cell infiltration but also on the regulation of immune checkpoint genes (Ruf et al., 2021). High *CAMSAP1* expression was positively related to immune checkpoints, in particular *CD274* (Rho >0.3, *p* < 0.001). *CD274*, also known as programmed cell death ligand 1 (PD-L1), is overexpressed in tumor cells to perform immune escape function by binding to PD-1 on the surface of CD8^+^ T cell (Hoos, 2016). Indeed, antibodies or drugs targeting PD-L1 have proven effective and well-tolerated for various cancers, including LIHC (Cheng et al., 2020). Moreover, our study implied that targeting *CAMSAP1* may reduce immune escape caused by PD-L1 and improve the immunotherapeutic effect elicited by anti-PD-L1 in LIHC patients. CAFs, another TME component, participate in immunosuppression, and promote tumor growth and survival by interacting with tumor cells, and are associated with poor patient outcomes (Mhaidly and Mechta-Grigoriou, 2021). Noteworthy, *CAMSAP1* was markedly correlated with CAFs but did not correlate with TMB or MSI in LIHC. ICB treatment prediction confirmed that compared with LIHC patients with high *CAMSAP1* expression, the low-expression group showed better treatment effect. Combined with clinical features, ICB treatment strategy was more effective for patients with early LIHC.

Taken together, *CAMSAP1* expression is associated with the diagnosis, progression, prognosis, and immunotherapy outcome of LIHC. During the initiation and malignant progression of LIHC, besides genetic alteration and DNA methylation, AC145207.5/LINC01748-miR-101-3p-mediated *CAMSAP1* overexpression induces LIHC with immune cell infiltration. However, immune checkpoints, mainly *CD274* (PD-L1), and CAFs are also found to be positively correlated with *CAMSAP1* expression, implying immune escape and a suppressed immune infiltrating microenvironment, thereby leading to poor prognosis. Targeting *CAMSAP1* may improve the immunotherapy effect of ICB treatment, thus induces favorable prognosis (Figure 7F). Our study provides novel insights for the further exploration of LIHC. Elucidating the potential role of *CAMSAP1* in immunotherapy will bring new hope for LIHC treatment, which is expected to become a personalized treatment option for patients with LIHC. However, further basic experiments and more clinical trials are required for these findings.

## Conclusion

In this study, for the first time, we demonstrate that besides genetic alteration and DNA methylation, AC145207.5/LINC01748-miR-101-3p-mediated *CAMSAP1* upregulation in advanced LIHC leads to poor prognosis with suppressed tumor immune infiltration, representing a potential diagnostic and prognostic biomarker as well as a promising immunotherapy target for LIHC.

## Data Availability

The datasets presented in this study can be found in online repositories. The names of the repository/repositories and accession number(s) can be found in the article/Supplementary Material.

## Glossary

LIHC: Liver hepatocellular carcinoma
MT: microtubule
CAMSAP: Calmodulin-regulated spectrin-associated protein
ncRNA: non-coding RNA
ICB: immune checkpoint blockade
microRNA: miRNA
lncRNA: long non-coding RNA
TCGA: The Cancer Genome Atlas
GTEx: Genotype-Tissue Expression
GEO: Gene Expression Omnibus
IHC: immunohistochemical
HPA: Human Protein Atlas
AOD: average optical density
CNA: copy-number alteration
lncRNA: long non-coding RNA
ENCORI: Encyclopedia of RNA Interactomes
ROC: receiver operating characteristic
AUC: area under the curve
OS: overall survival
DSS: disease-specific survival
PFI: progression-free interval
KM: Kaplan–Meier
OR: odds ratio
CI: confidence interval
HR: hazard ratio
C-index: concordance index
DEG: differentially expressed gene
GO: Gene Ontology
GSEA: Gene Set Enrichment Analysis
CC: cellular component
MF: molecular function
BP: biological pathway
TIMER2: Tumor Immune Estimation Resource 2
CAFs: cancer-associated fibroblasts
TMB: tumor mutation burden
MSI: microsatellite instability
TIDE: Tumor Immune Dysfunction and Exclusion
CTL: cytotoxic T lymphocyte
ACC: adrenocortical carcinoma
BLCA: bladder urothelial carcinoma
BRCA: breast infiltrating carcinoma
CESC: cervical squamous cell carcinoma and endocervical adenocarcinoma
CHOL: cholangiocarcinoma
COAD: colon adenocarcinoma
DLBC: diffuse large B cell lymphoma
ESCA: esophageal carcinoma
GBM: glioblastoma multiforme
HNSC: head and neck squamous cell carcinoma
KICH: kidney chromophobe
KIRC: kidney renal clear cell carcinoma
KIRP: kidney renal papillary cell carcinoma
LAML: acute myeloid leukemia
LGG: low-grade glioma
LUAD: lung adenocarcinoma
LUSC: lung squamous cell carcinoma
MESO: mesothelioma
OV: ovarian serous cystadenocarcinoma
PAAD: pancreatic adenocarcinoma
PCPG: pheochromocytoma and paraganglioma
PRAD: prostate adenocarcinoma
READ: rectum adenocarcinoma
SARC: sarcoma
SKCM: skin cutaneous melanoma
STAD: stomach adenocarcinoma
TGCT: testicular germ cell tumors
THCA: thyroid carcinoma
THYM: thymoma
UCEC: uterine corpus endometrial carcinoma
UCS: uterine carcinosarcoma
UVM: uveal melanoma
ceRNA: competing endogenous RNA

## References

[B1] Ahn J. C. Teng P. C. Chen P. J. Posadas E. Tseng H. R. Lu S. C. (2021). Detection of circulating tumor cells and their implications as a biomarker for diagnosis, prognostication, and therapeutic monitoring in hepatocellular carcinoma. Hepatology 73 (1), 422–436. 10.1002/hep.31165 32017145 PMC8183673

[B2] Anastasiadou E. Jacob L. S. Slack F. J. (2018). Non-coding RNA networks in cancer. Nat. Rev. Cancer 18 (1), 5–18. nature12986./nrc.2017.99 29170536 10.1038/nrc.2017.99 PMC6337726

[B3] Atherton J. Luo Y. Xiang S. Yang C. Rai A. Jiang K. (2019). Structural determinants of microtubule minus end preference in CAMSAP CKK domains. Nat. Commun. 10 (1), 5236. nature12986./s41467-019-13247-6 31748546 10.1038/s41467-019-13247-6 PMC6868217

[B4] Bai S. W. Herrera-Abreu M. T. Rohn J. L. Racine V. Tajadura V. Suryavanshi N. (2011). Identification and characterization of a set of conserved and new regulators of cytoskeletal organization, cell morphology and migration. BMC Biol. 9, 54. 10.1186/1741-7007-9-54 21834987 PMC3201212

[B5] Baines A. J. Bignone P. A. King M. D. Maggs A. M. Bennett P. M. Pinder J. C. (2009). The CKK domain (DUF1781) binds microtubules and defines the CAMSAP/ssp4 family of animal proteins. Mol. Biol. Evol. 26 (9), 2005–2014. 10.1093/molbev/msp115 19508979

[B6] Bonneville R. Krook M. A. Kautto E. A. Miya J. Wing M. R. Chen H. Z. (2017). Landscape of microsatellite instability across 39 cancer types. JCO Precis. Oncol. 2017, 1–15. 10.1200/PO.17.00073 PMC5972025 29850653

[B7] Chen Y. Zheng J. Li X. Zhu L. Shao Z. Yan X. (2020). Wdr47 controls neuronal polarization through the camsap family microtubule minus-end-binding proteins. Cell. Rep. 31 (3), 107526. 10.1016/j.celrep.2020.107526 32320668

[B8] Cheng A. L. Hsu C. Chan S. L. Choo S. P. Kudo M. (2020). Challenges of combination therapy with immune checkpoint inhibitors for hepatocellular carcinoma. J. Hepatol. 72 (2), 307–319. 10.1016/j.jhep.2019.09.025 31954494

[B9] Dolcino M. Tinazzi E. Puccetti A. Lunardi C. (2019). Long non-coding RNAs target pathogenetically relevant genes and pathways in rheumatoid arthritis. Cells 8 (8), E816. 10.3390/cells8080816 31382516 PMC6721587

[B10] Frost F. G. Cherukuri P. F. Milanovich S. Boerkoel C. F. (2020). Pan-cancer RNA-seq data stratifies tumours by some hallmarks of cancer. J. Cell. Mol. Med. 24 (1), 418–430. 10.1111/jcmm.14746 31730267 PMC6933344

[B11] Gao B. Bataller R. (2011). Alcoholic liver disease: Pathogenesis and new therapeutic targets. Gastroenterology 141 (5), 1572–1585. 10.1053/j.gastro.2011.09.002 21920463 PMC3214974

[B12] Goodson H. V. Jonasson E. M. (2018). Microtubules and microtubule-associated proteins. Cold Spring Harb. Perspect. Biol. 10 (6), a022608. 10.1101/cshperspect.a022608 29858272 PMC5983186

[B13] Hendershott M. C. Vale R. D. (2014). Regulation of microtubule minus-end dynamics by CAMSAPs and Patronin. Proc. Natl. Acad. Sci. U. S. A. 111 (16), 5860–5865. 10.1073/pnas.1404133111 24706919 PMC4000804

[B14] Hiam-Galvez K. J. Allen B. M. Spitzer M. H. (2021). Systemic immunity in cancer. Nat. Rev. Cancer 21 (6), 345–359. nature12986./s41568-021-00347-z 33837297 10.1038/s41568-021-00347-z PMC8034277

[B15] Hindson J. (2021). Molecular landscape of NASH-HCC. Nat. Rev. Gastroenterol. Hepatol. 18 (7), 456. nature12986./s41575-021-00478-6 10.1038/s41575-021-00478-6 34103708

[B16] Hoadley K. A. Yau C. Wolf D. M. Cherniack A. D. Tamborero D. Ng S. (2014). Multiplatform analysis of 12 cancer types reveals molecular classification within and across tissues of origin. Cell. 158 (4), 929–944. 10.1016/j.cell.2014.06.049 25109877 PMC4152462

[B17] Hoos A. (2016). Development of immuno-oncology drugs - from CTLA4 to PD1 to the next generations. Nat. Rev. Drug Discov. 15 (4), 235–247. nature12986./nrd.2015.35 26965203 10.1038/nrd.2015.35

[B18] Izzi V. Davis M. N. Naba A. (2020). Pan-cancer analysis of the genomic alterations and mutations of the matrisome. Cancers (Basel) 12 (8), E2046. 10.3390/cancers12082046 32722287 PMC7463652

[B19] Jackson J. R. Patrick D. R. Dar M. M. Huang P. S. (2007). Targeted anti-mitotic therapies: Can we improve on tubulin agents? Nat. Rev. Cancer 7 (2), 107–117. nature12986./nrc2049 17251917 10.1038/nrc2049

[B20] Jiang K. Faltova L. Hua S. Capitani G. Prota A. E. Landgraf C. (2018). Structural basis of formation of the microtubule minus-end-regulating CAMSAP-katanin complex. Structure 26 (3), 375–382. 10.1016/j.str.2017.12.017 29395789

[B21] Jiang K. Hua S. Mohan R. Grigoriev I. Yau K. W. Liu Q. (2014). Microtubule minus-end stabilization by polymerization-driven CAMSAP deposition. Dev. Cell. 28 (3), 295–309. 10.1016/j.devcel.2014.01.001 24486153

[B22] Jordan M. A. Wilson L. (2004). Microtubules as a target for anticancer drugs. Nat. Rev. Cancer 4 (4), 253–265. nature12986./nrc1317 15057285 10.1038/nrc1317

[B23] Kimanya M. E. Routledge M. N. Mpolya E. Ezekiel C. N. Shirima C. P. Gong Y. Y. (2021). Estimating the risk of aflatoxin-induced liver cancer in Tanzania based on biomarker data. PLoS One 16 (3), e0247281. 10.1371/journal.pone.0247281 33705417 PMC7951873

[B24] King M. D. Phillips G. W. Bignone P. A. Hayes N. V. Pinder J. C. Baines A. J. (2014). A conserved sequence in calmodulin regulated spectrin-associated protein 1 links its interaction with spectrin and calmodulin to neurite outgrowth. J. Neurochem. 128 (3), 391–402. 10.1111/jnc.12462 24117850 PMC4016758

[B25] Lee H. Kong S. W. Park P. J. (2008). Integrative analysis reveals the direct and indirect interactions between DNA copy number aberrations and gene expression changes. Bioinformatics 24 (7), 889–896. 10.1093/bioinformatics/btn034 18263644 PMC2600603

[B26] Lee Y. T. Wang J. J. Luu M. Tseng H. R. Rich N. E. Lu S. C. (2021). State-level HCC incidence and association with obesity and physical activity in the United States. Hepatology 74 (3), 1384–1394. 10.1002/hep.31811 33728665

[B27] Li D. Ding X. Xie M. Huang Z. Han P. Tian D. (2020a). CAMSAP2-mediated noncentrosomal microtubule acetylation drives hepatocellular carcinoma metastasis. Theranostics 10 (8), 3749–3766. 10.7150/thno.42596 32206120 PMC7069094

[B28] Li J. H. Liu S. Zhou H. Qu L. H. Yang J. H. (2014). starBase v2.0: decoding miRNA-ceRNA, miRNA-ncRNA and protein-RNA interaction networks from large-scale CLIP-Seq data. Nucleic Acids Res. 42, D92–D97. Database issue). 10.1093/nar/gkt1248 24297251 PMC3964941

[B29] Li S. Shao J. Lou G. Wu C. Liu Y. Zheng M. (2021). MiR-144-3p-mediated dysregulation of EIF4G2 contributes to the development of hepatocellular carcinoma through the ERK pathway. J. Exp. Clin. Cancer Res. 40 (1), 53. 10.1186/s13046-021-01853-6 33526055 PMC7852102

[B30] Li T. Fu J. Zeng Z. Cohen D. Li J. Chen Q. (2020b). TIMER2.0 for analysis of tumor-infiltrating immune cells. Nucleic Acids Res. 48 (W1), W509–W514. 10.1093/nar/gkaa407 32442275 PMC7319575

[B31] Liu J. Lichtenberg T. Hoadley K. A. Poisson L. M. Lazar A. J. Cherniack A. D. (2018). An integrated TCGA pan-cancer clinical data resource to drive high-quality survival outcome analytics. Cell. 173 (2), 400–416. 10.1016/j.cell.2018.02.052 29625055 PMC6066282

[B32] Liu X. Y. Li Y. Ji K. K. Zhu J. Ling P. Zhou T. (2020). Genome-wide codon usage pattern analysis reveals the correlation between codon usage bias and gene expression in Cuscuta australis. Genomics 112 (4), 2695–2702. 10.1016/j.ygeno.2020.03.002 32145379

[B33] Love M. I. Huber W. Anders S. (2014). Moderated estimation of fold change and dispersion for RNA-seq data with DESeq2. Genome Biol. 15 (12), 550. 10.1186/s13059-014-0550-8 25516281 PMC4302049

[B34] Lundberg Bave A. Bergquist A. Bottai M. Warnqvist A. Von Seth E. Nordenvall C. (2021). Increased risk of cancer in patients with primary sclerosing cholangitis. Hepatol. Int. 15, 1174–1182. 10.1007/s12072-021-10214-6 34357546 PMC8514354

[B35] Marcucci F. Rumio C. (2021). The tumor-promoting effects of the adaptive immune system: A cause of hyperprogressive disease in cancer? Cell. Mol. Life Sci. 78 (3), 853–865. 10.1007/s00018-020-03606-8 32940721 PMC11072297

[B36] Men C. Chai H. Song X. Li Y. Du H. Ren Q. (2017). Identification of DNA methylation associated gene signatures in endometrial cancer via integrated analysis of DNA methylation and gene expression systematically. J. Gynecol. Oncol. 28 (6), e83. 10.3802/jgo.2017.28.e83 29027401 PMC5641533

[B37] Mhaidly R. Mechta-Grigoriou F. (2021). Role of cancer-associated fibroblast subpopulations in immune infiltration, as a new means of treatment in cancer. Immunol. Rev. 302 (1), 259–272. 10.1111/imr.12978 34013544 PMC8360036

[B38] Olynyk J. K. Ramm G. A. (2021). Risk of liver cancer in HFE-hemochromatosis. Gastroenterology 161, 1718–1719. 10.1053/j.gastro.2021.08.025 34419460

[B39] Pawlotsky J. M. (2004). Pathophysiology of hepatitis C virus infection and related liver disease. Trends Microbiol. 12 (2), 96–102. 10.1016/j.tim.2003.12.005 15036326

[B40] Qi X. Zhang D. H. Wu N. Xiao J. H. Wang X. Ma W. (2015). ceRNA in cancer: possible functions and clinical implications. J. Med. Genet. 52 (10), 710–718. 10.1136/jmedgenet-2015-103334 26358722

[B41] Reinfeld B. I. Madden M. Z. Wolf M. M. Chytil A. Bader J. E. Patterson A. R. (2021). Cell-programmed nutrient partitioning in the tumour microenvironment. Nature 593 (7858), 282–288. nature12986./s41586-021-03442-1 33828302 10.1038/s41586-021-03442-1 PMC8122068

[B42] Richardson C. E. Spilker K. A. Cueva J. G. Perrino J. Goodman M. B. Shen K. (2014). PTRN-1, a microtubule minus end-binding CAMSAP homolog, promotes microtubule function in *Caenorhabditis elegans* neurons. Elife 3, e01498. 10.7554/eLife.01498 24569477 PMC3932522

[B43] Ruf B. Heinrich B. Greten T. F. (2021). Immunobiology and immunotherapy of HCC: Spotlight on innate and innate-like immune cells. Cell. Mol. Immunol. 18 (1), 112–127. nature12986./s41423-020-00572-w 33235387 10.1038/s41423-020-00572-w PMC7852696

[B44] Sheng Y. Li J. Zou C. Wang S. Cao Y. Zhang J. (2014). Downregulation of miR-101-3p by hepatitis B virus promotes proliferation and migration of hepatocellular carcinoma cells by targeting Rab5a. Arch. Virol. 159 (9), 2397–2410. 10.1007/s00705-014-2084-5 24788845

[B45] Skvortsova K. Stirzaker C. Taberlay P. (2019). The DNA methylation landscape in cancer. Essays Biochem. 63 (6), 797–811. 10.1042/EBC20190037 31845735 PMC6923322

[B46] Strassburg S. Nabar N. Lampert F. Goerke S. M. Pfeifer D. Finkenzeller G. (2017). Calmodulin regulated spectrin associated protein 1 mRNA is directly regulated by miR-126 in primary human osteoblasts. J. Cell. Biochem. 118 (7), 1756–1763. 10.1002/jcb.25838 27958650

[B47] Sun W. Zhang Q. Wu Z. Xue N. (2019). miR-101-3p sensitizes hepatocellular carcinoma cells to oxaliplatin by inhibiting Beclin-1-mediated autophagy. Int. J. Clin. Exp. Pathol. 12 (6), 2056–2065. 31934027 PMC6949619

[B48] Sun X. Wang Z. M. Song Y. Tai X. H. Ji W. Y. Gu H. (2014). MicroRNA-126 modulates the tumor microenvironment by targeting calmodulin-regulated spectrin-associated protein 1 (Camsap1). Int. J. Oncol. 44 (5), 1678–1684. 10.3892/ijo.2014.2321 24603804

[B49] Sung H. Ferlay J. Siegel R. L. Laversanne M. Soerjomataram I. Jemal A. (2021). Global cancer statistics 2020: GLOBOCAN estimates of incidence and mortality worldwide for 36 cancers in 185 countries. Ca. Cancer J. Clin. 71 (3), 209–249. 10.3322/caac.21660 33538338

[B50] Tan Y. Xu F. Xu L. Cui J. (2022). Long noncoding RNA LINC01748 exerts carcinogenic effects in nonsmall cell lung cancer cell lines by regulating the microRNA520a5p/HMGA1 axis. Int. J. Mol. Med. 49 (2), 22. 10.3892/ijmm.2021.5077 34970695 PMC8722766

[B51] Tay Y. Rinn J. Pandolfi P. P. (2014). The multilayered complexity of ceRNA crosstalk and competition. Nature 505 (7483), 344–352. 10.1038/nature12986 24429633 PMC4113481

[B52] Thorsson V. Gibbs D. L. Brown S. D. Wolf D. Bortone D. S. Ou Yang T. H. (2018). The immune landscape of cancer. Immunity 48 (4), 812–830. e814. 10.1016/j.immuni.2018.03.023 29628290 PMC5982584

[B53] Trepo C. Chan H. L. Lok A. (2014). Hepatitis B virus infection. Lancet 384 (9959), 2053–2063. 10.1016/S0140-6736(14)60220-8 24954675

[B54] Vivian J. Rao A. A. Nothaft F. A. Ketchum C. Armstrong J. Novak A. (2017). Toil enables reproducible, open source, big biomedical data analyses. Nat. Biotechnol. 35 (4), 314–316. 10.1038/nbt.3772 28398314 PMC5546205

[B55] Wang H. W. Hsieh T. H. Huang S. Y. Chau G. Y. Tung C. Y. Su C. W. (2013). Forfeited hepatogenesis program and increased embryonic stem cell traits in young hepatocellular carcinoma (HCC) comparing to elderly HCC. BMC Genomics 14, 736. 10.1186/1471-2164-14-736 24160375 PMC3826595

[B56] Wattanathamsan O. Chetprayoon P. Chantaravisoot N. Wongkongkathep P. Chanvorachote P. Pongrakhananon V. (2021). CAMSAP3 depletion induces lung cancer cell senescence-associated phenotypes through extracellular signal-regulated kinase inactivation. Cancer Med. 10 (24), 8961–8975. 10.1002/cam4.4380 34724356 PMC8683528

[B57] Wu M. Wang Y. Liu H. Song J. Ding J. (2020). Genomic analysis and clinical implications of immune cell infiltration in gastric cancer. Biosci. Rep. 40 (5), BSR20193308. 10.1042/BSR20193308 32338286 PMC7240200

[B58] Xu F. Yan J. J. Gan Y. Chang Y. Wang H. L. He X. X. (2019). miR-885-5p negatively regulates Warburg effect by silencing hexokinase 2 in liver cancer. Mol. Ther. Nucleic Acids 18, 308–319. 10.1016/j.omtn.2019.09.002 31614321 PMC6796743

[B59] Yi L. Wu G. Guo L. Zou X. Huang P. (2020). Comprehensive analysis of the PD-L1 and immune infiltrates of m(6)A RNA methylation regulators in head and neck squamous cell carcinoma. Mol. Ther. Nucleic Acids 21, 299–314. 10.1016/j.omtn.2020.06.001 32622331 PMC7332506

[B60] Yi Y. Qiu Z. Yao Z. Lin A. Qin Y. Sha R. (2021). CAMSAP1 mutation correlates with improved prognosis in small cell lung cancer patients treated with platinum-based chemotherapy. Front. Cell. Dev. Biol. 9, 770811. 10.3389/fcell.2021.770811 35087829 PMC8787262

[B61] Yu G. Wang L. G. Han Y. He Q. Y. (2012). clusterProfiler: an R package for comparing biological themes among gene clusters. OMICS 16 (5), 284–287. 10.1089/omi.2011.0118 22455463 PMC3339379

[B62] Zhou Z. Xu H. Li Y. Yang M. Zhang R. Shiraishi A. (2020). CAMSAP1 breaks the homeostatic microtubule network to instruct neuronal polarity. Proc. Natl. Acad. Sci. U. S. A. 117 (36), 22193–22203. 10.1073/pnas.1913177117 32839317 PMC7486724

