# Supplementary material for: Non-coding ribonucleic acid-mediated CAMSAP1 upregulation leads to poor prognosis with suppressed immune infiltration in liver hepatocellular carcinoma
Source: Front Genet. 2022 Sep 21;13:916847. doi: 10.3389/fgene.2022.916847 (PMC9532701; doi:10.3389/fgene.2022.916847)
Supplement: Supplementary file 1 [file DataSheet1.ZIP › Supplementary Materials/Supplementary Materials.docx]

# SUPPLEMENTARY MATERIALS

## SUPPLEMENTARY FIGURES

### FIGURE S1 | *CAMSAP1* promoter methylation level in normal and primary tumor from UALCAN portal. Related to Figure 2E. *p* > 0.05 was considered no significant difference.

### FIGURE S2 | *CAMSAP1*-associated prognosis in upregulated and downregulated cancer patients. Related to Figure 3C. (A-D) KM analyses of OS, DSS, and PFI from TCGA database with significant differences (A, B) and no significant difference (C, D). Cox regression analysis was used. *p* < 0.05 was considered statistically significant.

### FIGURE S3 | Immunohistochemical images of CAMSAP1 proteins in normal (A) and LIHC (B) tissues from the HPA database. Related to Figure 4B and C. Scare bar, 200 μm.

### FIGURE S4 | Overexpressed *CAMSAP1* predicts poor DSS in advanced LIHC. Related to Figure 4F-H. (A) A forest plot of univariate and multivariate Cox regression analysis with DSS. * *p* < 0.05, ** *p* < 0.01, *** *p* < 0.001. (B) A nomogram for predicting the probability of 1-, 3-, and 5-years DSS. (C) Nomogram calibration analysis with DSS.

### FIGURE S5 | Overexpressed *CAMSAP1* predicts poor PFI in advanced LIHC. Related to Figure 4F-H. (A) A forest plot of univariate and multivariate Cox regression analysis with PFI. * *p* < 0.05, ** *p* < 0.01, *** *p* < 0.001. (B) A nomogram for predicting the probability of 1-, 3-, and 5-years PFI. (C) Nomogram calibration analysis with PFI.

### FIGURE S6 | Relationship between *CAMSAP1* and immune-associated genes in LIHC from TCGA dataset. Related to Figure 7C and D. (A-D) Scatter plots of correlation between *CAMSAP1* and immune marker genes (A), immune checkpoints (B), TMB score (C), and MSI score (D). *p* < 0.05 was considered statistically significant.

## SUPPLEMENTARY TABLES

### Table S1 | Correlation between *CAMSAP1* expression and CNAs from cBioPortal (TCGA, Pancancer Atlas). Related to Figure 2D.

### Table S2 | Correlation between *CAMSAP1* expression and DNA methylation from cBioPortal (TCGA, Firehose). Related to Figure 2F.

### Table S3 | Correlation between *CAMSAP1* and predicted miRNAs in LIHC from ENCORI portal. Related to Figure 5A.

### Table S4 | Correlation between hsa-miR-101-3p and predicted lncRNAs in LIHC from ENCORI portal. Related to Figure 5G.

**Table S5 |** *CAMSAP1*-related top20 DEGs and their correlation from TCGA database. Related to Figure 6B.

### Table S6 | GO analysis of *CAMSAP1*-related DEGs. Related to Figure 6C.

### Table S7 | GSEA analysis of *CAMSAP1*-related DEGs. Related to Figure 6D-G.

### Table S8 | Relationship between *CAMSAP1* and immune cell markers in LIHC from TIMER2 and TCGA. Related to Figure 7C.

### Table S9 | Relationship between *CAMSAP1* and immune-regulated genes in LIHC from TIMER2 and TCGA. Related to Figure 7D.

### Table S10 | *CAMSAP1*-associated ICB therapeutic effect in 25% low and high *CAMSAP1* expression groups from TCGA database using TIDE algorithm. Related to Figure 7E.
