# Supplementary figures and images for: Non-coding ribonucleic acid-mediated CAMSAP1 upregulation leads to poor prognosis with suppressed immune infiltration in liver hepatocellular carcinoma
Source: Front Genet. 2022 Sep 21;13:916847. doi: 10.3389/fgene.2022.916847 (PMC9532701; doi:10.3389/fgene.2022.916847)

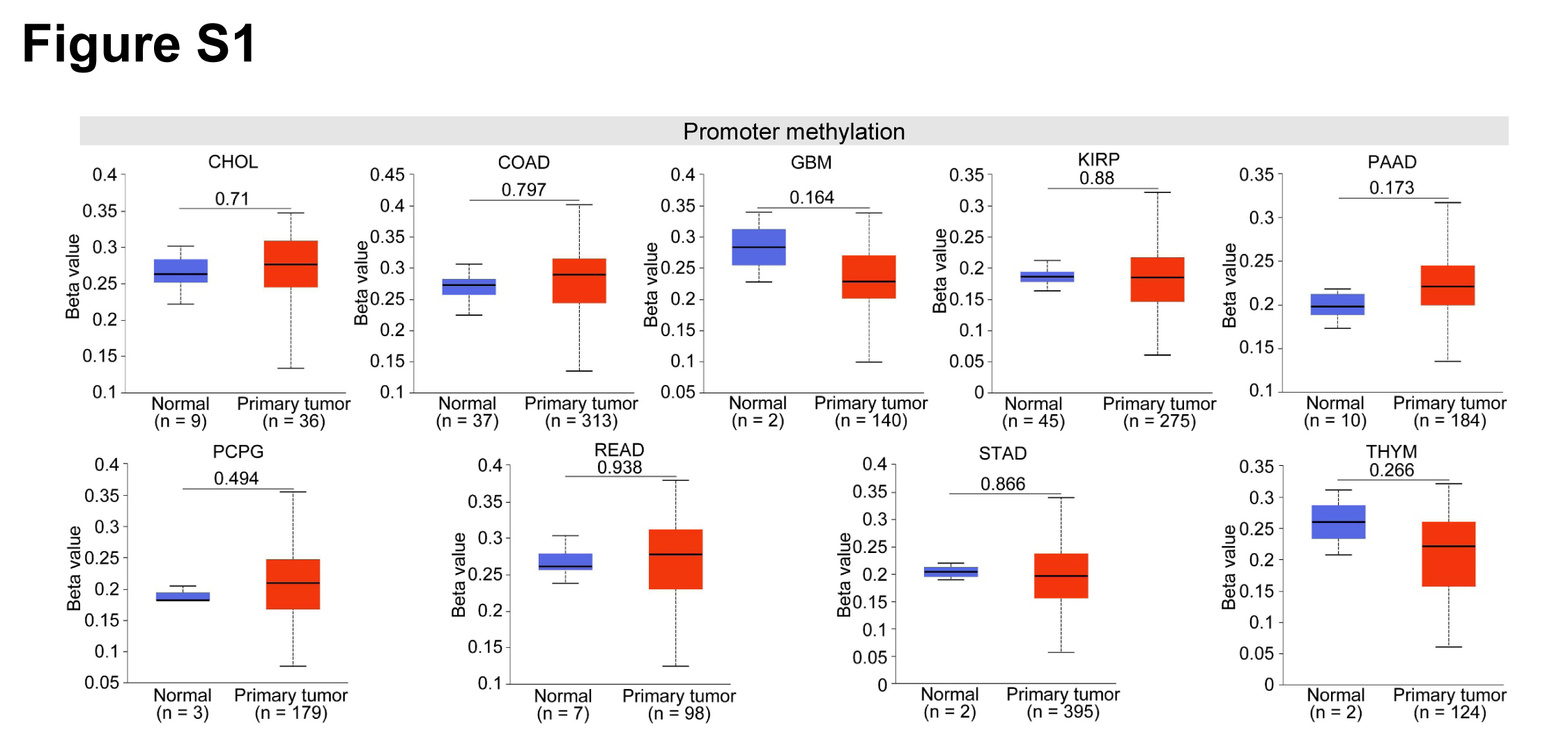

Supplement: Supplementary file 1 [file DataSheet1.ZIP › Supplementary Materials/Supplementary Figures 1. CAMSAP1 promoter methylation level in normal and primary tumor from UALCAN portal..tif]

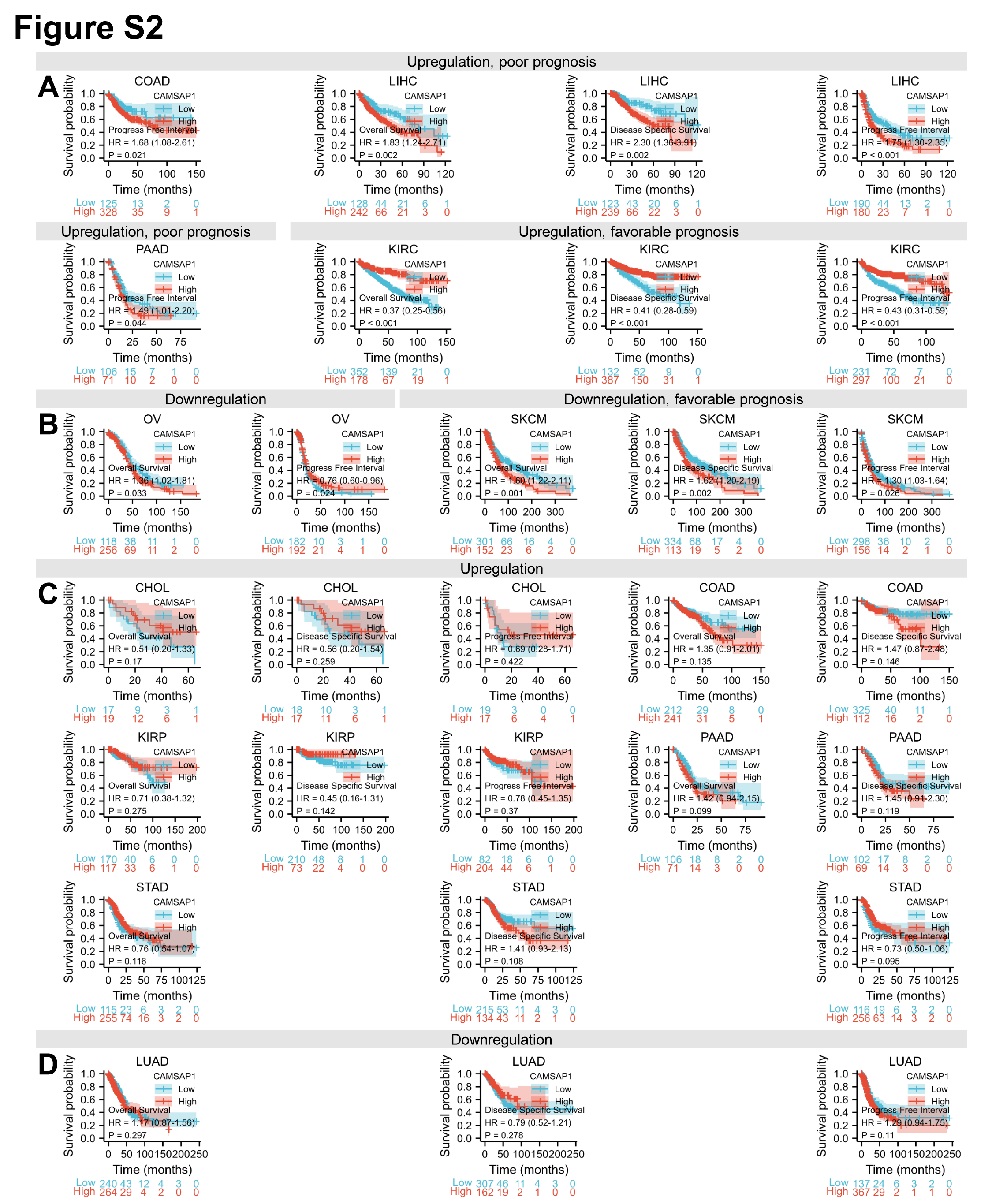

Supplement: Supplementary file 1 [file DataSheet1.ZIP › Supplementary Materials/Supplementary Figures 2. CAMSAP1-associated prognosis in upregulated and downregulated cancer patients..tif]

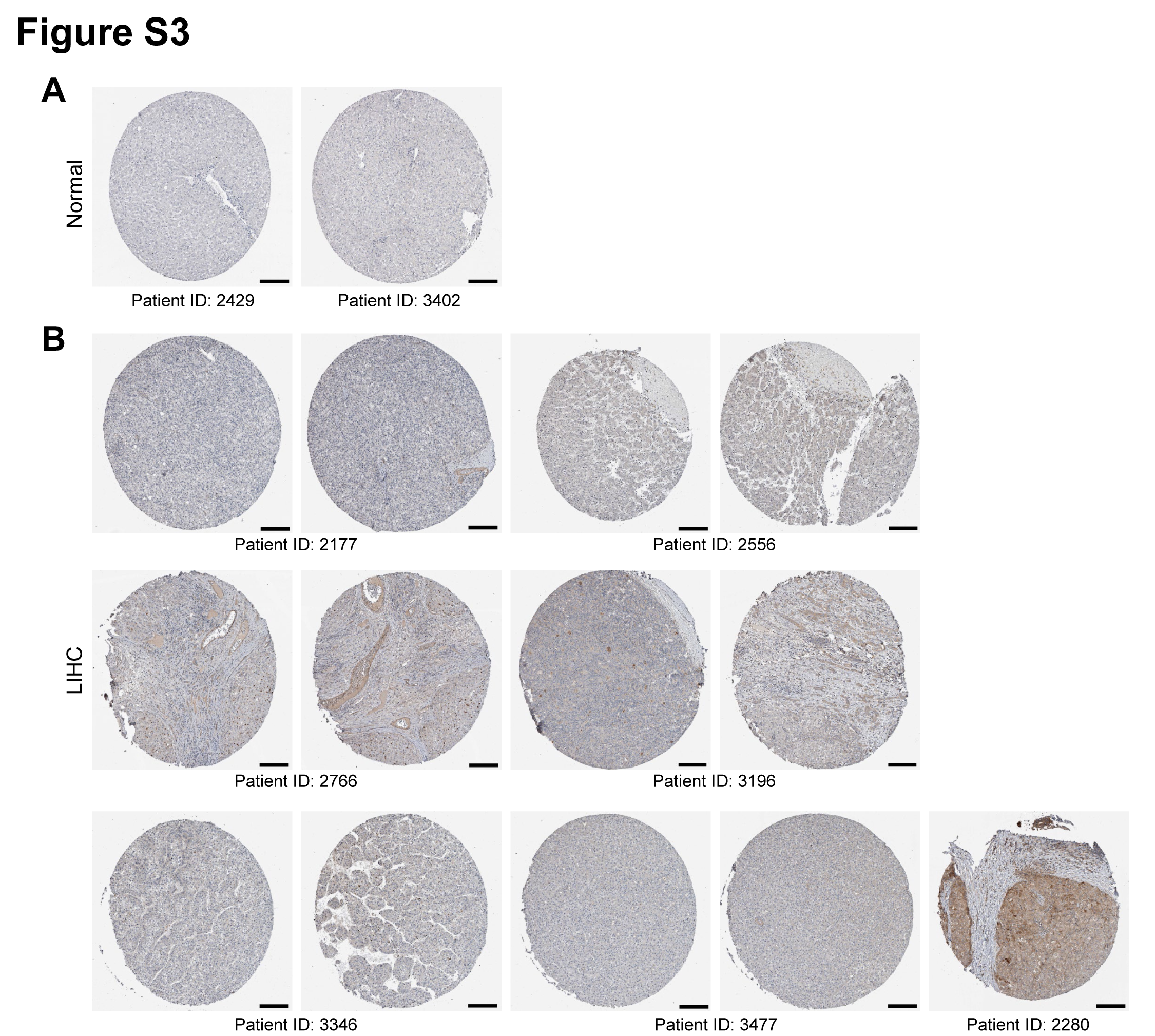

Supplement: Supplementary file 1 [file DataSheet1.ZIP › Supplementary Materials/Supplementary Figures 3. Immunohistochemical images of CAMSAP1 proteins in normal (A) and LIHC (B) tissues from HPA database..tif]

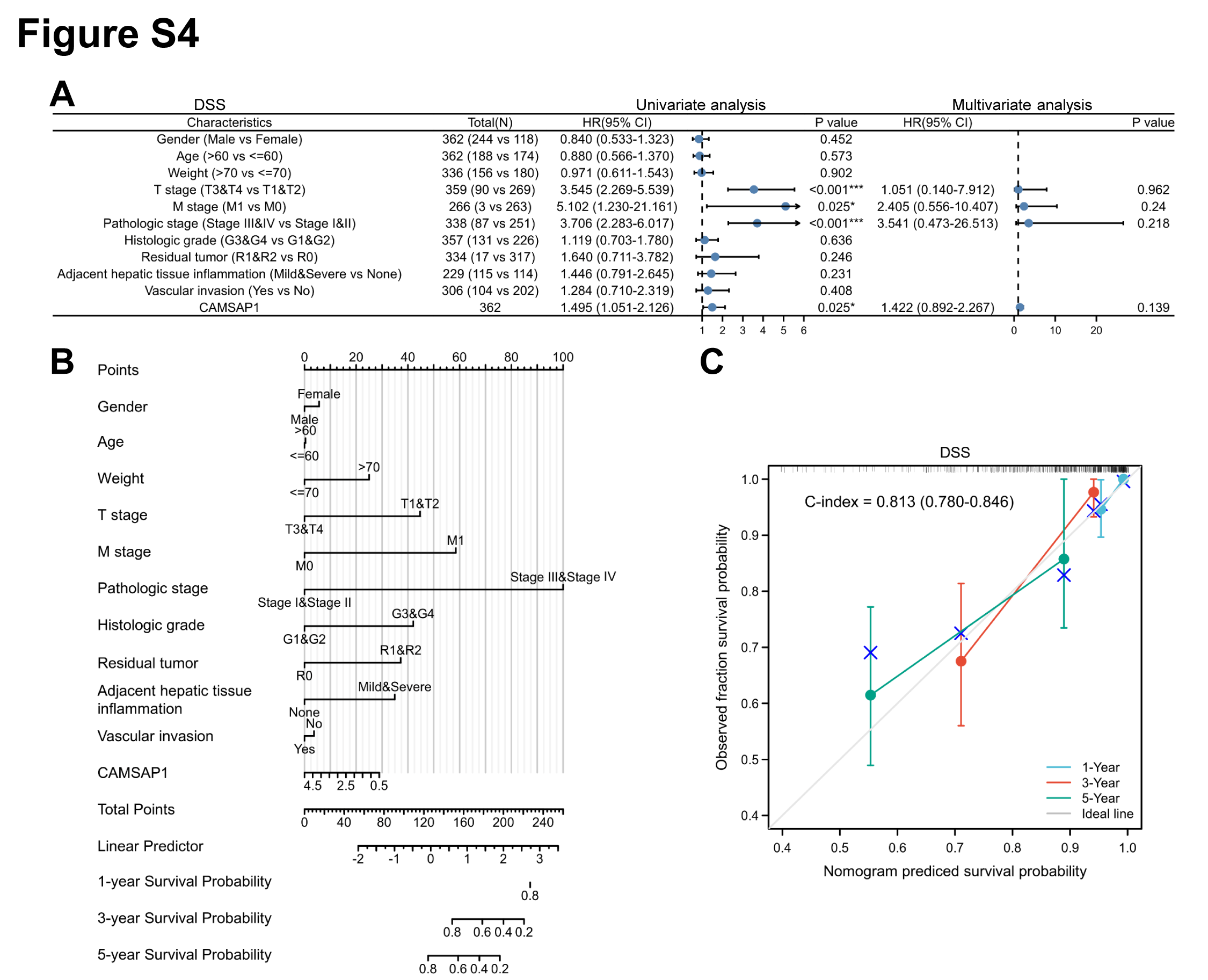

Supplement: Supplementary file 1 [file DataSheet1.ZIP › Supplementary Materials/Supplementary Figures 4. Overexpressed CAMSAP1 predicts poor DSS in advanced LIHC..tif]

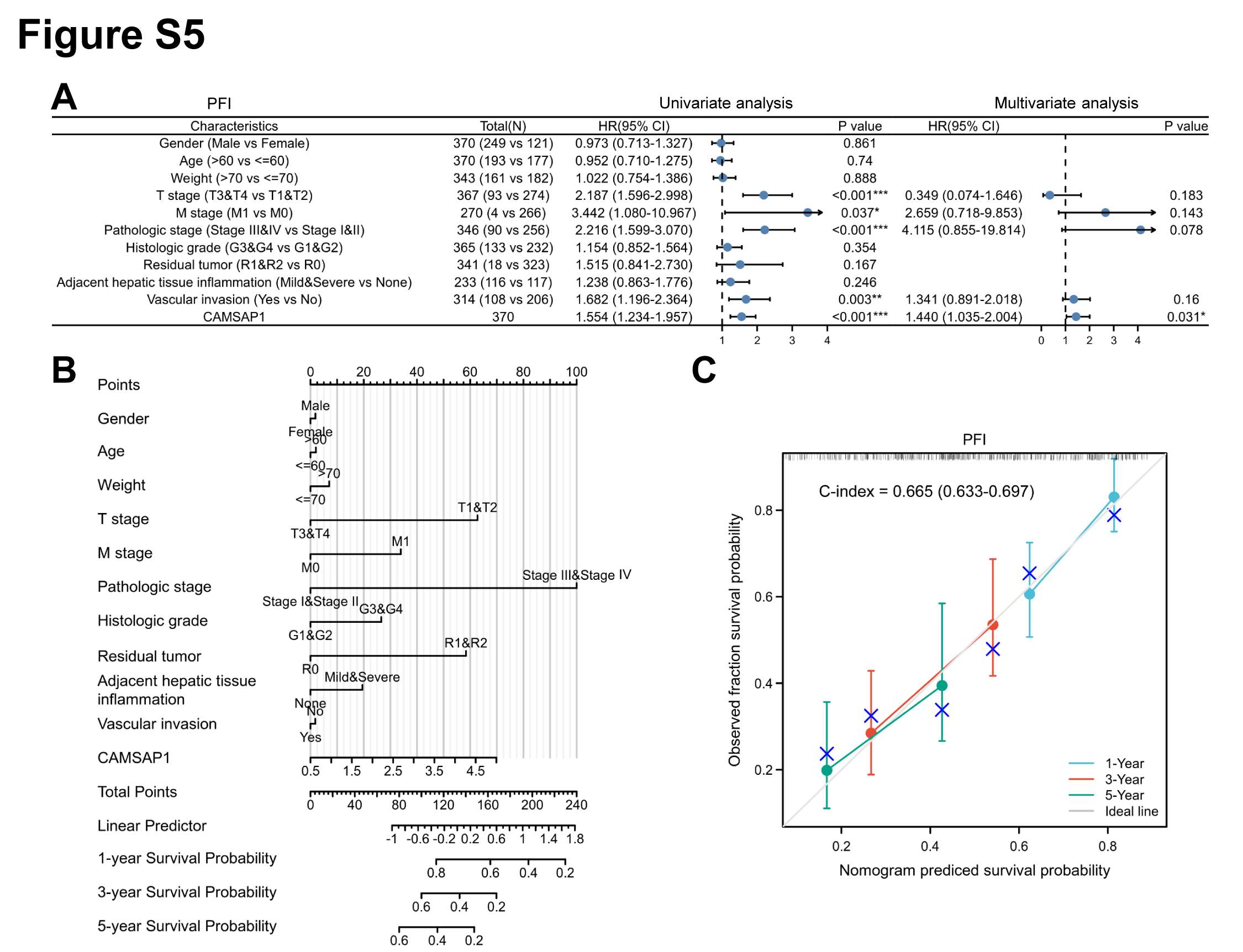

Supplement: Supplementary file 1 [file DataSheet1.ZIP › Supplementary Materials/Supplementary Figures 5. Overexpressed CAMSAP1 predicts poor PFI in advanced LIHC..tif]

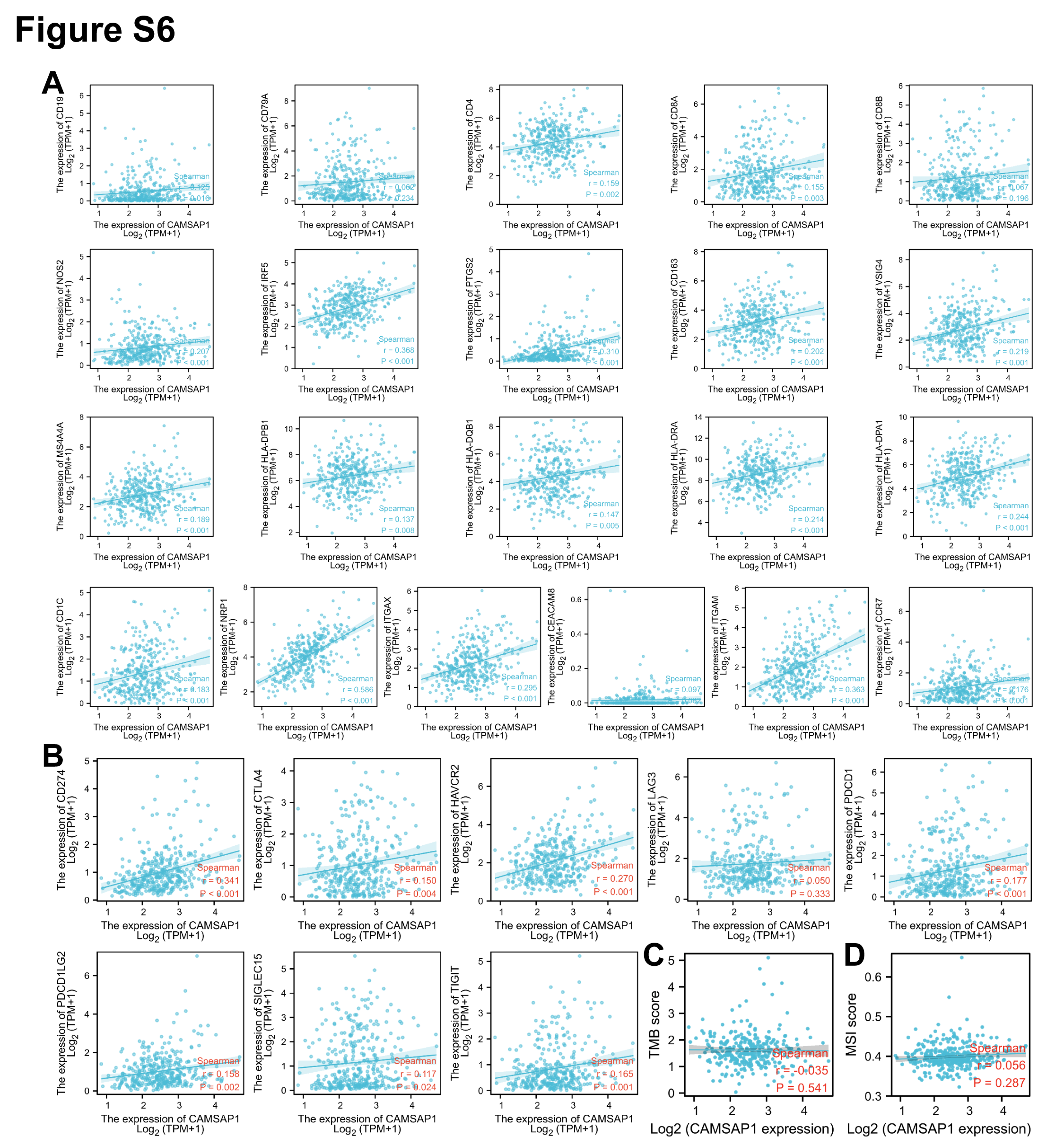

Supplement: Supplementary file 1 [file DataSheet1.ZIP › Supplementary Materials/Supplementary Figures 6. Relationship between CAMSAP1 and immune-associated genes in LIHC from TCGA dataset..tif]
